# Advanced Materials for NH_3_ Capture: Interaction Sites and Transport Pathways

**DOI:** 10.1007/s40820-024-01425-1

**Published:** 2024-06-27

**Authors:** Hai-Yan Jiang, Zao-Ming Wang, Xue-Qi Sun, Shao-Juan Zeng, Yang-Yang Guo, Lu Bai, Ming-Shui Yao, Xiang-Ping Zhang

**Affiliations:** 1 Key Laboratory of Green Process and Engineering, State Key Laboratory of Mesoscience and Engineering, Beijing Key Laboratory of Ionic Liquids Clean Process, Institute of Process Engineering, Chinese Academy of Sciences, Beijing, 100190 People’s Republic of China; 2 School of Future Technology, University of Chinese Academy of Sciences, Beijing, 100049 People’s Republic of China; 3 China University of Petroleum, Beijing, 102249 People’s Republic of China; 4 Institute for Integrated Cell-Material Sciences (iCeMS), Kyoto University, Sakyo-Ku, YoshidaKyoto, 606-8501 Japan

**Keywords:** Ammonia capture, Solvents, Porous solids, Porous liquids, Membranes

## Abstract

Ammonia (NH_3_) is a carbon-free, hydrogen-rich chemical related to global food safety, clean energy, and environmental protection. As an essential technology for meeting the requirements raised by such issues, NH_3_ capture has been intensively explored by researchers in both fundamental and applied fields. The four typical methods used are (1) solvent absorption by ionic liquids and their derivatives, (2) adsorption by porous solids, (3) ab-adsorption by porous liquids, and (4) membrane separation. Rooted in the development of advanced materials for NH_3_ capture, we conducted a coherent review of the design of different materials, mainly in the past 5 years, their interactions with NH_3_ molecules and construction of transport pathways, as well as the structure–property relationship, with specific examples discussed. Finally, the challenges in current research and future worthwhile directions for NH_3_ capture materials are proposed.

## Highlights

- An overview of advanced materials for NH_3_ capture from the aspects of interaction sites and transport pathways is presented.
- The classifications, working principles, design ideas and structure–property relationships on materials for NH_3_ capture are discussed in detail.
- The challenges and encouraging outlooks with worthwhile directions for NH_3_ capture are proposed.

## Graphical abstract

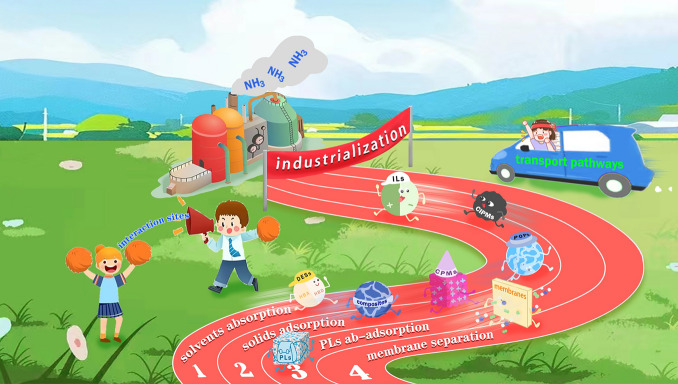

## Introduction

Ammonia (NH_3_), an important basic chemical, is a feed stock for nitrogenous fertilizer production via the Haber process, which is important for global food safety [1–3]. It is also a promising clean energy source owing to its high hydrogen density and carbon-free nature, and it provides safer transport and storage compared with H_2_ due to its easy liquefaction and low penetration rate toward transport equipment [4–8]. However, NH_3_ is a toxic and irritating gas that is detrimental to human health. Specifically, it injures the human eyes, skin, respiratory tract, and liver when its concentration in the blood is higher than 25 ppm [9]. Meanwhile, the excessive emission of NH_3_ in the atmosphere will participate in chemical reactions to form 2.5-µm particulate matter (PM2.5), causing negative effects such as haze and soil acidification, etc. [10, 11].

NH_3_-containing gases come from a wide range of sources. For example, it is inevitable to generate a large amount of NH_3_-containing exhausted gas during urea manufacturing and ammonia synthesis processes. In addition to the mentioned chemical process, the direct NH_3_ emission from agriculture such as compost and animal breed place also causes serious negative effects [11, 12]. Therefore, NH_3_ capture and recovery from these sources benefit both resource utilization and environmental protection. The traditional technologies for capturing NH_3_ involve physical condensation and water/acid scrubbing. Physical condensation relies on a boiling point difference to achieve separation. In such a case, NH_3_-containing gas should be cooled to a lower temperature (e.g., − 15 °C) to liquify gaseous NH_3_, while other compounds remain gaseous, which always consumes more energy. Water/acid scrubbing depends on the different solubilities of gases in liquid solvents to achieve gas separation. However, the NH_3_ recovery from water is energy-intensive, and large quantities of NH_3_-containing wastewater are inevitably produced, causing serious secondary pollution. Inorganic acid solutions, such as H_2_SO_4_ and H_3_PO_4_ are highly corrosive, and the reaction of inorganic acids with NH_3_ is almost irreversible and generates the low-valued salts.

Based on the above analysis, it is necessary to develop novel green technologies for NH_3_ capture and recovery, in which the design and controllable fabrication of advanced materials are crucial. To date, many materials have been developed, including ionic liquids (ILs), crystalline porous materials (CPMs), porous organic polymers (POPs), and their composites. However, most reviews have focused on a single topic such as ILs for NH_3_ absorption [13–15] or metal–organic frameworks (MOFs) for NH_3_ adsorption [16–19]. Overall reviews of both developed and emerging NH_3_ capture materials are still limited. Rooted in the development of the advanced materials for NH_3_ capture, we aimed to provide a coherent review of the design of different materials mainly over the past 5 years, and their interactions with NH_3_ molecules, and the construction of transport pathways. This review first presents a summary of the categories of materials, including functional solvents, porous solids, porous liquids and emerging membranes, along with brief working principles and evaluated parameters. Then, the recent advancements in such materials are briefly reviewed in detail. Functional solvents including ILs and deep eutectic solvents (DESs), have been introduced due to their structural tunability, negligible vapor pressure, and lower energy consumption compared with traditional solvents. As for various NH_3_-containing gases separation system, balancing the absorption–desorption ability, costs, and variations in physical properties of functional solvents is challenging. As an alternative strategy, porous solids involving conventional inorganic porous materials (CIPMs), porous organic polymers (POPs), crystalline porous materials (CPMs), and composite adsorbents have been proposed, and their performances have been analyzed based on the pore properties and type of interaction sites. Such solids are difficult to be implemented in conventional flow processes, and their performance remains limited. And most of them faced with the problem of structural collapse. Based on the fluidity of liquid absorbents and the porosity of porous solids, an important direction for porous liquids (PLs) for NH_3_ ab-adsorption was proposed. However, this technology is on the rise and not yet mature and requires to further development. Emerging organic, inorganic and hybrid membranes for NH_3_ separation and their gas separation performance are discussed; however, it is difficult to meet the demands of industrialization, and the long-term stability of various membranes has still not been explored. In the conclusions and prospects of this review, challenges in current research and encouraging outlooks for the future application of such materials in advanced NH_3_ capture are analyzed and proposed.

## Working Principles

The design and development of these materials are important for achieving efficient NH_3_ capture. Excellent NH_3_-capturing materials require two features. One is rich specific sites that can interact with NH_3_ molecules to attain high affinity. It should be noted that the interaction cannot be too strong; otherwise, it is not conducive to the release of captured NH_3_ from the materials. The other is the introduction of transport pathways, which are expected to provide modulable diffusion channels and rich accessible sites, thereby improving the NH_3_ capture performance and reducing regeneration consumption.

**Fig. 1:**
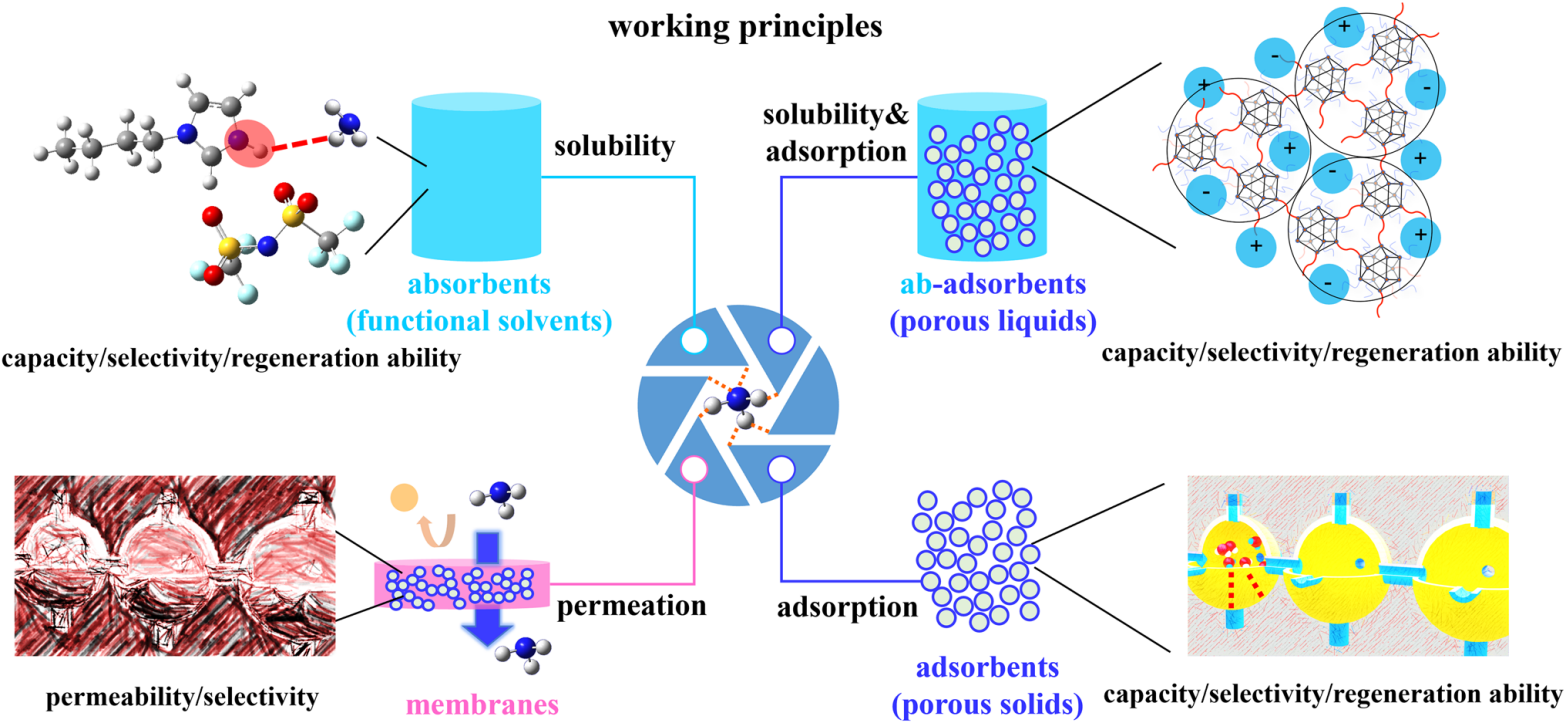
Working principles of NH_3_ capture materials

According to the material characteristics and capture principle, the NH_3_ capture materials can be divided into the following four types as shown in Fig. 1: absorbents (functional solvents, Sect. 3), adsorbents (porous solids, Sect. 4), ab-adsorbents (porous liquids, Sect. 5), and membrane materials (Sect. 6).

Functional solvents utilize gas with different solubilities in liquid solvents to achieve selective NH_3_ absorption. The interaction sites (hydroxyl groups, acidic protons, amino groups, metal ions, etc.) on functional solvents play an important role in enhancing the NH_3_ absorption performance. The NH_3_ absorption capacity of a given solvent, which is largely influenced by the pressure and temperature, can be determined by the gravimetric methods, vapor–liquid equilibrium apparatus, etc. [20, 21]. The regeneration ability of absorbents is another important evaluation parameter that is significantly related to the energy efficiency and economic benefits in practical applications.

The working principle of porous solids relies mainly on their confined micropores to accommodate gas molecules and the interaction sites in these pores to achieve selective NH_3_ adsorption. The pore structures and interaction site strengths of porous solids can be obtained using a physical adsorption apparatus, temperature-programmed desorption of ammonia, and other methods. The NH_3_ adsorption isotherm is normally measured using a gas adsorption instrument that monitors the change in pressure of a sample held at a given temperature when exposed to different ammonia pressures [22]. The NH_3_ adsorption dynamics of samples can be investigated either by breakthrough curves [16], which record the concentration curve of each component over time through a breakthrough column, or by dynamic mode measured on a gas adsorption instrument [23], which can provide the speed to reach equilibrium and the time-dependent adsorption capacity at a given pressure and temperature.

Although porous solids offer major benefits, such as lower energy penalties in adsorption–desorption cycles, they are difficult to implement in conventional flow processes. To address this limitation, ab-adsorbents, i.e., PLs, have been developed by introducing permanent porosity into liquid materials. The existence of intrinsic micropores in PLs allows for rapid NH_3_ adsorption–desorption (kinetics) while maintaining liquid fluidity and high adsorption capacity and selectivity (thermodynamics) resulting from both components. Such a combination is also beneficial for reducing the regeneration consumption and thus increasing the energy efficiency compared with liquid absorption, owing to the introduction of a pore structure on the feasible gas diffusion pathways [24]. The gas uptake of PLs can be measured by gas adsorption equipment [25] and column breakthrough tests [26]. Annihilation lifetime spectroscopy (PALS) and density measurements [27, 28] are usually used to confirm the permanent porosity of PLs.

Membrane separation uses different gas permeation rates through membranes to achieve NH_3_ selective separation. Gas permeation tests usually use the differential pressure method; specifically, they can be divided into the constant pressure-variable volume and constant volume-variable pressure methods. Permeance and selectivity are key parameters for gas separation membranes [29, 30]. The permeance (*P*_*i*_) and separation selectivity ($\alpha_{i/j}$) can be calculated using the following equations:

1 $$P_{i} = \frac{{Q_{i} }}{{A\Delta p_{i} }}$$

2 $$\alpha_{i/j} = \frac{{P_{i} }}{{P_{j} }}$$

where *P*_*i*_ and *P*_*j*_ represent the permeance of gases *i* and *j*, respectively (cm^3^ (STP)/(cm^2^ s cm Hg)); 1 GPU = 1 × 10^–6^ cm^3^ (STP)/(cm^2^ s cm Hg); *Q*_*i*_ denotes permeate flow rate of gas *i* at the standard state (cm^3^ (STP) s^−1^); and *A* and Δ*p*_i_ represent the effective membrane area (cm^2^) and transmembrane pressure difference of gas *i*, respectively.

## Functional Solvents for NH_3_ Absorption

**Fig. 2:**
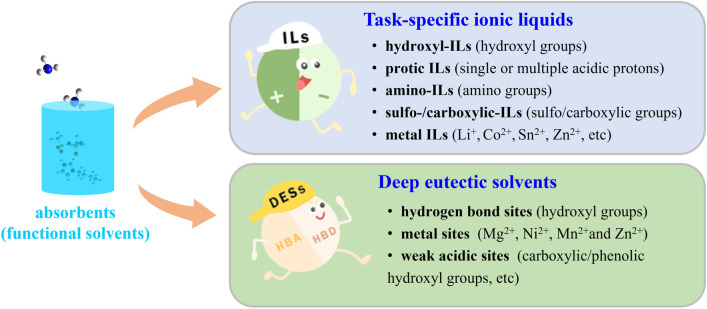
Interaction sites on functional solvents for NH_3_ absorption

As advanced solvents, ionic liquids (ILs) are prospective candidates for NH_3_ capture. ILs are entirely composed of organic cations and organic/inorganic anions, which make them designable according to application requirements [13–15]. In addition, the unique properties of ILs, including negligible vapor pressure, low specific heat capacity, and excellent recyclability, greatly reduce the regeneration energy consumption and solvent loss during the NH_3_ capture process compared to water scrubbing [15, 31]. Current research on NH_3_ capture using IL-based solvents involves the design and development of absorbents, mass-transfer investigation, process simulation and assessment, and industrial applications. The development of task-specific absorbents for efficient and reversible NH_3_ capture is fundamental and critical; thus, it has attracted the attention of many researchers. In this section, the NH_3_ absorption–desorption performance, physical property variation, and absorption mechanism of task-specific ILs and their analogous DESs are briefly discussed from the perspective of the types and numbers of interaction sites for NH_3_ absorption (Fig. 2).

### Task-Specific Ionic Liquids

Considerable efforts have been devoted to designing novel task-specific ILs for efficient NH_3_ absorption. The interaction sites between ILs and NH_3_ molecules play an important role in efficient and reversible NH_3_ absorption. The introduction of hydroxyl groups, acidic protons, amino groups, sulfo-/carboxyl groups, and metal ions remarkably improves the NH_3_ absorption capacity of ILs*.* Table 1 lists the NH_3_ absorption capacities of representative ILs.

**Table 1:** NH_3_ absorption capacity of representative ILs

ILs	*T* (K)	*P* (kPa)	NH_3_ absorption capacity		References
			(mol NH_3_/mol IL)	(mg NH_3_/g IL)	
[Emim][BF_4_]	298	140	0.282	24	[34]
[Hmim][BF_4_]	298	220	0.485	32	[34]
[Omim][BF_4_]	298	120	0.389	23	[34]
[Bmim][SCN]	303	145	0.320	28	[34]
[Bmim][NTf_2_]	313	101	0.280	11	[38]
[Emim][Ac]	298.2	463	1.506	151	[32]
[Emim][EtOSO_3_]	298.1	421	1.075	77	[32]
[Emim][SCN]	298.1	307	0.799	80	[32]
[Bim][NTf_2_]	313	101	2.690	113	[38]
[Choline][NTf_2_]	293	100	1.857	82	[34]
[MTEOA][MeOSO_3_]	293	101	3.545	232	[35]
[DMEA][Ac]	298.1	278	1.604	183	[32]
[EtOHmim][NTf_2_]	313	128	0.830	35	[36]
[EtOHim][NTf_2_]	313	100	3.110	135	[21]
[EtOHim][BF_4_]	313	100	2.470	210	[21]
[EtOHim][SCN]	313	100	2.230	222	[21]
[MeOHim][NTf_2_]	313	100	3.040	136	[21]
[2-Mim][NTf_2_]	313	101	3.037	142	[41]
[Im]][NTf_2_]	313	101	3.461	169	[41]
[1, 2, 3-TrizH_2_][NO_3_]_2_	313	101	4.187	365	[20]
[Eim][Li(NTf_2_)_2_]	313	101	6.618	169	[41]
[2-Mim][Li(NTf_2_)_2_]	313	101	7.012	183	[41]
[EtA][SCN]	293	101	2.538	359	[45]
[Bmim][Zn_2_Cl_5_]	323	103.5	8.025	305	[47]
[Emim]_2_[Co(NCS)_4_]	303	101	5.990	178	[49]
[Bmim]_2_[Co(NCS)_4_]	303	101	6.030	163	[49]
[Hmim]_2_[Co(NCS)_4_]	303	101	6.090	151	[49]
[Bmim]_2_[CuCl_4_]	303	101	4.611	172	[50]
[Bmim]_2_[NiCl_4_]	343	101	4.559	195	[50]
[Bmim]_2_[SnCl_4_]	303	101	5.169	108	[50]
[Li-TEG][NTf_2_]	313	102.5	3.36	131	[51]

The dissolution behavior of NH_3_ in conventional ILs was firstly reported in 2007, and it was inferred that strong intermolecular complexes between NH_3_ and ILs are formed [32]. Subsequently, it was found that cations had a greater influence on NH_3_ solubility than anions and that the hydrogen bond between the acidic 2-H of the imidazole cation and N atom of NH_3_ played a crucial role in NH_3_ absorption [33]. Thus, a feasible strategy for designing task-specific ILs is to tune the hydrogen-donating ability of the cations by adjusting the type and number of functional groups.

**Fig. 3:**
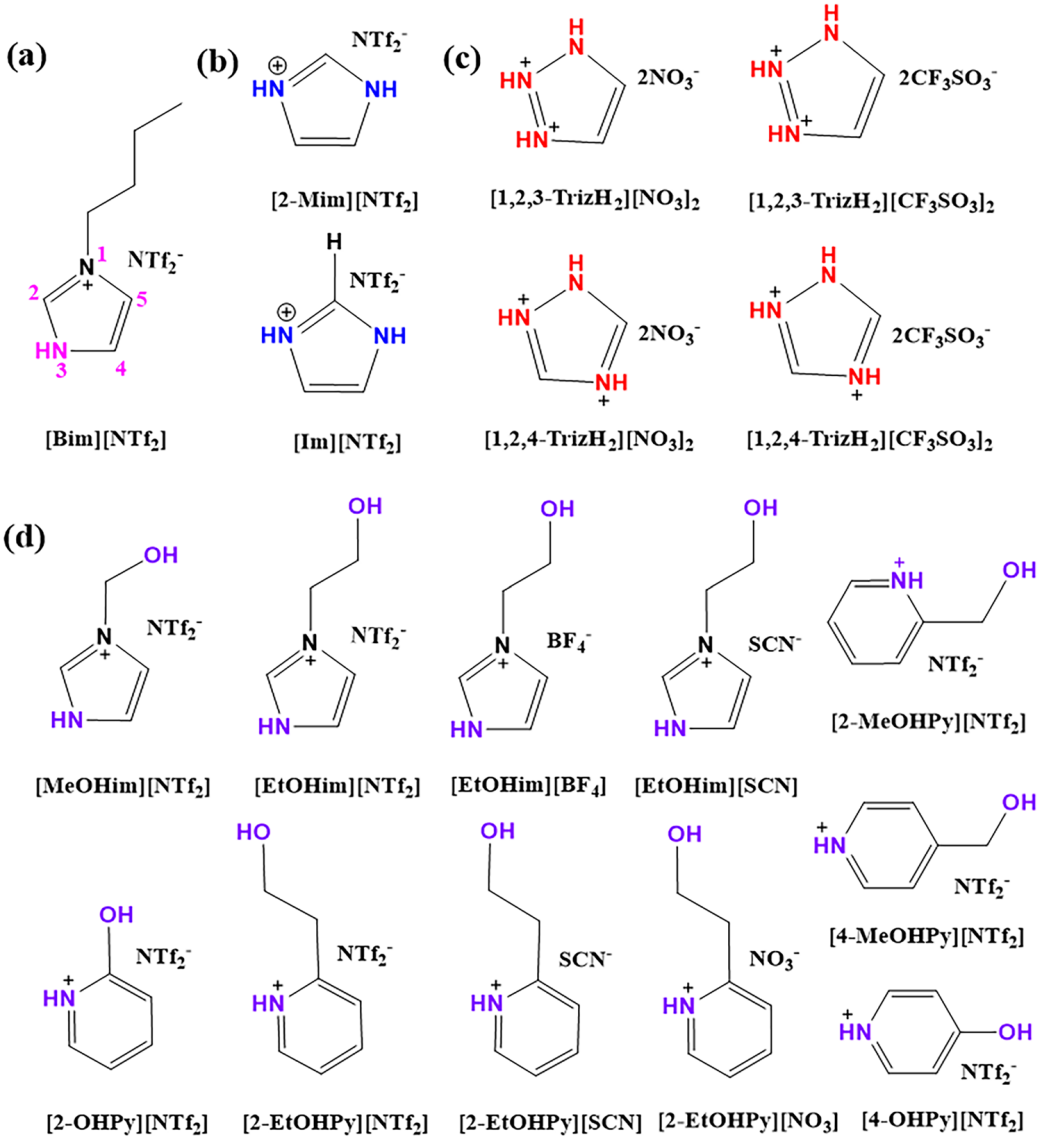
**a** Structures of [Bim][NTf_2_] [38]. **b** [2-Mim][NTf_2_] and [Im][NTf_2_] [41]. **c** Triazole cation-functionalized ILs [20]. **d** Dual-functionalized protic ILs [21, 42]

A series of task-specific ILs based on the hydrogen bond interaction were developed by introducing single hydroxyl functional groups and acidic protons into cations of ILs, and these ILs usually have higher NH_3_ absorption capacity (0.83–4.2 mol NH_3_/mol IL) than that of conventional ILs (< 0.8 mol NH_3_/mol IL), simultaneously showing great regeneration ability. Palomar et al. [34, 35] adopted the COSMO-RS calculation method to screen potential NH_3_ absorbents from 272 ILs and found that hydroxyl-functionalized ILs [EtOHmim][BF_4_] and [choline][NTf_2_] are promising for NH_3_ absorption. As expected, a higher absorption capacity was achieved by hydroxyl-functionalized ILs compared with conventional ILs, and the separation mechanism of hydrogen bond interactions between ILs and NH_3_ was further proved by near-infrared spectroscopy (NIR) and nuclear magnetic resonance (NMR) spectroscopy [36, 37]. Shang et al. [38] proposed a new strategy for introducing an acidic proton onto a cation to improve the NH_3_ absorption capacity. The protic ionic liquid (PIL) [Bim][NTf_2_] (Fig. 3a) exhibited high NH_3_ absorption capacity with a value of 2.69 mol NH_3_/mol IL at 313 K and 100 kPa. There was no evident decline in the absorption ability of the recycled [Bim][NTf_2_] after being used four times. Notably, the viscosity of the IL–NH_3_ system during the NH_3_ absorption process first increased and then decreased sharply to a lower value [39], which is completely different from the increased viscosity caused by CO_2_ absorption. Furthermore, the NH_3_ absorption mechanism of ILs was revealed through molecular dynamics (MD) simulations [40]. The results indicated that the energy of strong N3–H···N(NH_3_) hydrogen bond between [Bim]^+^ and NH_3_ molecules is up to − 79.0 kJ mol^−1^, which is twice as strong as the hydrogen bond energy between C2-H of [Bmim]^+^ and NH_3_. This strong interaction induced the enrichment of cations at the PIL–gas interface, resulting in NH_3_ molecules penetrating deeply into the bulk of the PILs and achieving selective absorption of NH_3_ from gases containing N_2_ and H_2_. Besides, there are always other gases present, such as water in NH_3_-containing gases in industrial streams. Trace water was also found to enhance NH_3_ absorption owing to the cooperative absorption caused by [Bim][NTf_2_] and H_2_O.

Multiple hydrogen sites can be incorporated into the cations of ILs to improve their absorption performance through cooperative hydrogen bonding interactions. The NH_3_ absorption capacity of imidazole-based ILs ([2-Mim][NTf_2_] and [Im][NTf_2_]) (Fig. 3b) with two acidic protons on cations was up to 3.46 mol NH_3_/mol IL. The NH_3_ absorption capacity remained stable after five cycles of absorption and desorption [41]. Subsequently, Sun et al. [20] synthesized triazole cation-functionalized ionic liquids (TCFILs) containing three acidic protons (Fig. 3c). These TCFILs showed a rapid transition from the initial solid to liquid state during the NH_3_ absorption process. Moreover, [1, 2, 3-TrizH_2_][NO_3_]_2_ exhibited an ultrahigh NH_3_ absorption capacity of 4.187 mol NH_3_/mol IL (about 365 mg NH_3_/g IL) at 303.15 K and 101 kPa and great recyclability benefiting from multiple hydrogen bonds, which is comparable to that of traditional water absorbents used in industry (300 mg NH_3_/g H_2_O at 313 K and 101 kPa). Meanwhile, [1, 2, 3-TrizH_2_][CF_3_SO_3_]_2_ showed faster absorption kinetics than that of [1, 2, 3-TrizH_2_][NO_3_]_2_. At the same time, the effect of water molecules on NH_3_ absorption performance was studied. The results indicated that the addition of small amounts of water to [1, 2, 3-TrizH_2_][CF_3_SO_3_]_2_ had no obvious impact on the NH_3_ capacity and shortened the absorption equilibrium time from 20 to 15 min, owing to the reduced viscosity of the systems. Additionally, simultaneously embedding acidic protons and hydroxyl groups on the cations of ILs is an efficient strategy to further improve NH_3_ absorption capacity. Yuan et al. [21, 42] found that these imidazole- and pyridinium-based dual-functionalized PILs (DPILs) (Fig. 3d) possessed higher NH_3_ solubility than ILs functionalized only by a single hydroxyl group. Specifically, the NH_3_ solubility of [EtOHim][NTf_2_] was as high as 3.110 mol NH_3_/mol IL, which is approximately 30-fold greater than that of [Emim][NTf_2_] and four-fold greater than that of the functionalized IL [EtOHmim][NTf_2_]. These DPILs also exhibited outstanding recyclability, an excellent NH_3_/CO_2_ selectivity of 65, and NH_3_/ N_2_ selectivity of 104.

**Fig. 4:**
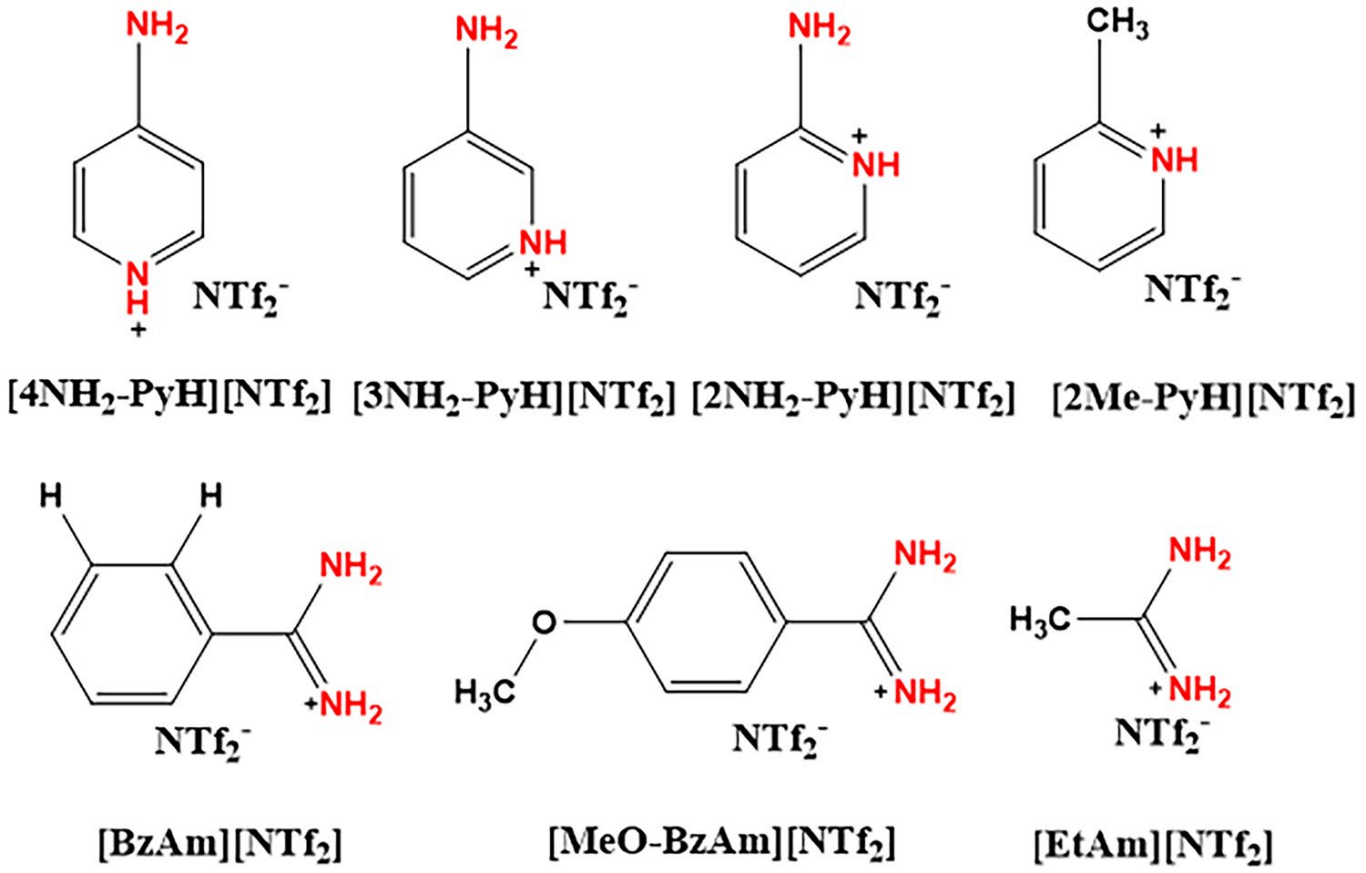
Structures of cation-functional PILs with single or multiple amidino-groups [43, 44]

There have been a few reports on the application of amino-functionalized ILs for NH_3_ absorption. For example, Luo et al. [43, 44] designed a series of cation-functional PILs with single or multiple amidino groups (Fig. 4). Reversible cooperative hydrogen bond (CHB) networks were formed by hydrogen bond interactions between ammonia and amidino groups. The NH_3_ absorption–desorption process was accompanied by the breakage and reformation of CHBs in the ILs, which led to a sigmoidal NH_3_ absorption isotherm and energy-saving desorption. The [BzAm][NTf_2_] showed NH_3_ absorption with a threshold pressure of 0.28 kPa and capacity of 2.8 mol NH_3_/mol IL at 100 kPa. The absorbed NH_3_ could also be rapidly stripped at 323 K and 1 kPa within 30 min. In addition, the threshold pressure and NH_3_ ammonia production capacity could be tuned by varying the CHB interactions in the ILs. Similarly, Deng et al. [45] synthesized six protic ethanolamine-based ILs with multiple binding sites for efficient and reversible NH_3_ uptake. Among them, ethanolamine thiocyanate ([EtA][SCN]) had suitable viscosity of 78.18 mPa s and exhibited the best absorption ability of 2.538 mol NH_3_/mol IL at atmospheric pressure and 293.15 K due to multiple hydrogen-bonding interactions between acidic protons, hydroxyl groups, and thiocyanate with NH_3_. In addition, the outstanding NH_3_/CO_2_ ideal selectivity with a value of 365 was observed in [EtA][SCN], which provides a competitive way to selectively separate NH_3_ from CO_2_ in tail gas.

At the same time, NH_3_ is typically an alkaline gas. The introduction of Brønsted acidic groups to react with NH_3_ is expected to improve the absorption capacity of ILs. Recent studies have shown that imidazolium- and ammonium-based ILs with sulfo and carboxy groups exhibit higher NH_3_ solubilities than conventional and hydroxy-functionalized ILs. Moreover, the acidity of the Brønsted acidic group and the chemical structures of the acidic group and constituent ions also significantly affected the NH_3_ capacity [46]. Another effective approach for improving the NH_3_ absorption capacity is to develop metal ILs based on complexation with NH_3_. Pioneering work on metal ILs for NH_3_ absorption was reported in 2013, which used [Bmim][Zn_2_Cl_5_] as an NH_3_ absorbents and showed a superior absorption capacity of 8.0 mol NH_3_/mol IL at 323 K and 100 kPa, but the strong complex interaction between metal ILs and NH_3_ molecules led to irreversibility of the materials [47, 48]. To solve the above problems, Zeng et al. [49] designed a series of novel cobalt ILs, [C_*n*_mim]_2_[Co(NCS)_4_] (*n* = 2, 4, or 6), for reversible NH_3_ absorption. The cobalt ILs exhibited a remarkable NH_3_ absorption capacity of 6.09 mol NH_3_/mol IL, which is more than 30 times higher than those of conventional ILs [C_*n*_mim][SCN] without metals. This superior performance was attributed to the moderate Lewis acid–base interaction and cooperative hydrogen bonding between the MILs and NH_3_ confirmed by experimental characterizations and density functional theory (DFT) calculations. At the same time, these cobalt ILs exhibited excellent recyclability and maintained a stable NH_3_ capacity after five cycles. Wang et al. [50] further systematically studied the effects of various metal centers on the physicochemical properties and NH_3_ absorption capacity. Among the range of MILs, [Bmim]_2_[SnCl_4_] not only showed a high absorption capacity of 5.169 mol NH_3_/mol IL at 303.15 K and 100 kPa, which is much higher than that of conventional ILs, but also showed no obvious NH_3_ capacity loss after five absorption and desorption cycles.

**Fig. 5:**
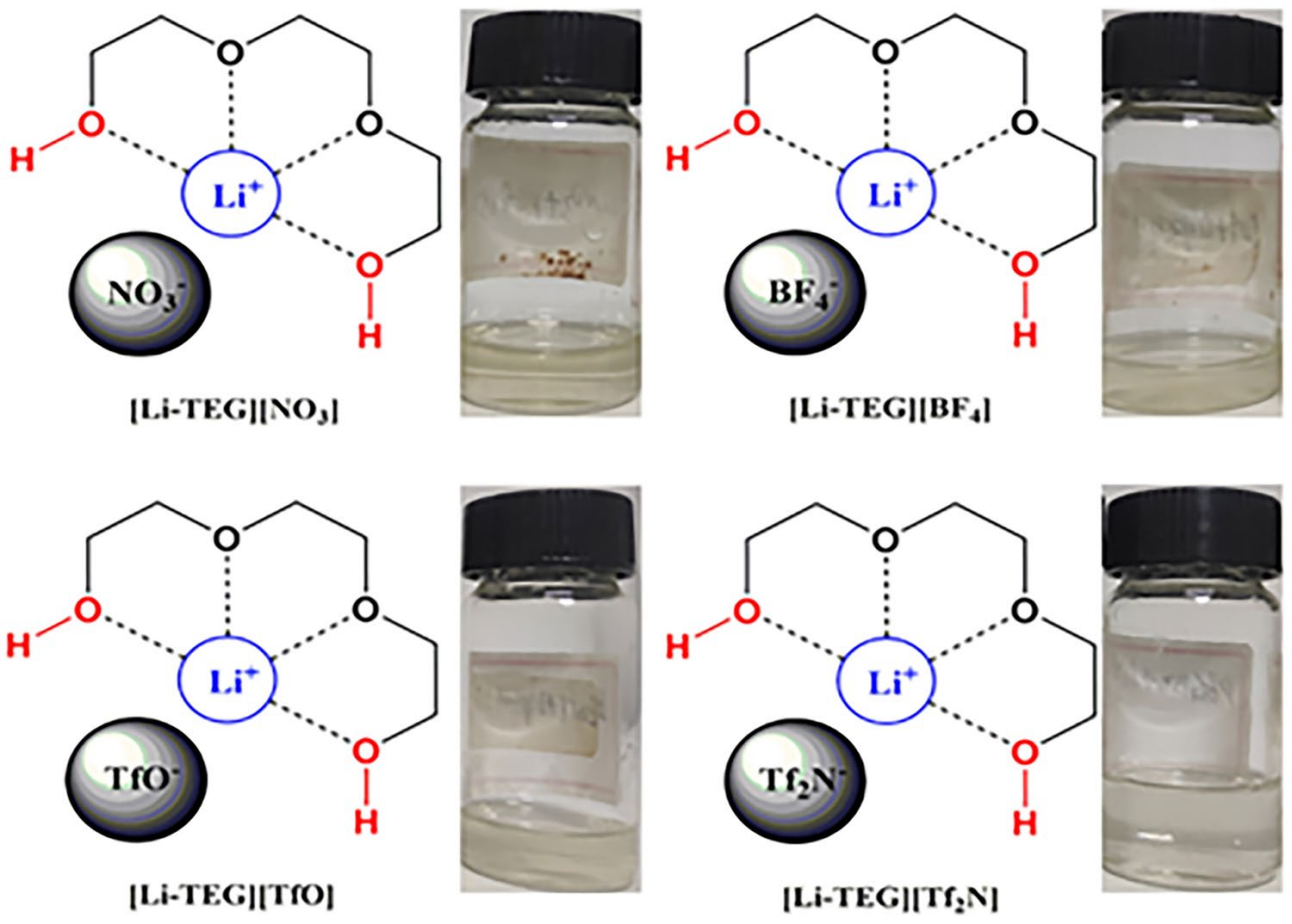
Structures and pictures of Li-TEG–chelated ILs. Reproduced with permission from Ref. [51], Copyright 2022, John Wiley and Sons

In addition to the above high-valence MILs, alkali metal ions, especially lithium (Li), have also been introduced into PILs to increase NH_3_ absorption performance. Shang et al. [41] prepared novel sorbents that simultaneously incorporate acidic protons into cations and Li^+^ ions into anions. The solid ILs gradually became liquids after NH_3_ adsorption. An exceptional NH_3_ capacity of 7.01 mol NH_3_/mol IL was achieved using [2-Mim][Li(NTf_2_)_2_] at 313 K and atmospheric pressure, which is the highest NH_3_ capacity reported for an IL to date. This superior capacity is attributed to the synergistic effect of hydrogen bonding between acidic protons and NH_3_, as well as the Lewis acid–base interaction between the Li^+^-based anion and NH_3_. Inspired by this, Cai et al. [51] further synthesized liquid chelation-activated multi-site ILs for reversible chemical absorption of NH_3_, as shown in Fig. 5. The chelation of triethylene glycol (TEG) with Li^+^ activates the hydroxyl sites in TEG for strong interaction with NH_3_, resulting into an outstanding NH_3_ absorption capacity of 3.36 mol NH_3_/mol IL at 313 K and 102.5 kPa.

### Deep Eutectic Solvents

Given the lone-pair electrons and alkalinity of NH_3_, the DESs with strong hydrogen-bond donating ability or Brøn-sted acidity are usually useful for capturing NH_3_. DESs generally consist of two or three components capable of intermolecular interactions, particularly hydrogen bond interactions, which have lower melting points than those of each separate component [52]. They can be easily prepared by simply mixing hydrogen bond acceptors (HBAs) with hydrogen bond donors (HBDs). The introduction of a second or third component effectively reduces the viscosity and improves the mass transfer efficiency. Because of the diverse structures of HBAs and HBDs, many DESs have been synthesized for NH_3_ absorption. Table 2 lists the NH_3_ absorption capacities of representative DESs.

**Table 2:** NH_3_ absorption capacity of representative DESs

DES	*T* (K)	*P* (kPa)	NH_3_ absorption capacity (mg NH_3_/g DES)	References
ChCl/Res/Gly (1:3:5)	313	101	130	[53]
ChCl/urea (1:2)	298	95	38	[54]
ChCl/1,4-BD (1:4)	303	115.8	57	[55]
MgCl_2_/Res/EG (0.1:1:2)	313	100	205	[56]
Tz/Gly (1:3)	303	101.3	179	[57]
[TMPDA][Cl]_2_/PhOH(1:7)	298	93.1	156	[58]
[Emim][Cl]/Tetz (1:1)	313	10.0	79	[59]
ChCl/EG (1:2)	313	100.5	46	[60]
ChCl/PhOH/EG (1:5:4)	298	101.3	164	[61]
ChCl/TetrZ/EG (3:7:14)	313	104.9	169	[62]
ChCl/xylose (1.5:1)	343	101.3	66	[63]
[MeC_3_^OH^N]Cl/EG (1:2)	313.2	101	73	[64]
[Me_2_C^OH^_2_C’^OH^N]Cl/Urea (1:1)	313.2	101.3	35.3	[65]
EaCl/AA (1:1)	313	96.4	65	[67]
EaCl/Gly (1: 2)	298	106.7	164	[68]
EaCl/PhOH (1:7)	298	101.3	85	[69]
EaCl/Res (1: 1)	298	101.2	182	[70]
3,4-DHAB + EG (1:3)	298	100	199	[71]
[Bmim][MeSO_3_]/urea (1:2)	313	172.6	18	[72]
[Im][NO_3_]/EG (1:3)	303	100	211	[73]
MAA/tetrazole (2:1)	313	102.9	136	[74]
KSCN/Gly (2:3)	313	100	101	[75]
NH_4_SCN/Gly(2:3)	303	100	223	[75]
GI/AT (1:2)	303	101	90	[76]

The current interest in DESs for NH_3_ absorption is rooted in the pioneering work on hybrid ternary DESs with flexible hydrogen-bonded supramolecular networks designed by Li et al. [53]. The reported DESs are composed of choline chloride (ChCl), resorcinol (Res), and glycerol (Gly), which break the trade-off between NH_3_-DES interaction strength and the stability of traditional DESs. The NH_3_ mass solubility of ChCl/Res/Gly (1:3:5) DESs reached 130 mg g^−1^ at 313 K and 101 kPa, which exceeds those of hydroxyl-functionalized ILs and ordinary DESs. More importantly, this excellent performance was retained after ten absorption–desorption cycles. Additionally, the presence of CO_2_ in melamine tail gases is unavoidable. Thus, the CO_2_ absorption of optimized DESs was investigated. The results showed that the solubility of CO_2_ was 0.91 mg g^−1^, which is far lower than NH_3_ solubility under the same conditions, showing great potential for the separation of NH_3_ and CO_2_.

Subsequently, a series of DESs using ChCl as the HBA were developed for NH_3_ capture owing to their excellent biodegradability and low price, including ChCl/Urea [54] and ChCl/dihydric alcohols [55]. Sun et al. [56] innovatively introduced metal chlorides, such as anhydrous MgCl_2_, NiCl_2_, MnCl_2_, and ZnCl_2_, into a binary Res/EG system to prepare ternary DESs, and the NH_3_ capacity notably increased owing to the cooperating hydrogen bonding and Lewis acid–base interactions. In particular, the NH_3_ absorption capacity of MgCl_2_/Res/EG (0.1:1:2) was 289 mg g^−1^ at 293 K and 100 kPa. In addition, the introduction of hydroxyl, amide, and carboxyl groups into the structure of HBDs in DESs was an effective method to obtain NH_3_ absorbents with excellent performance [57–59]. ChCl-based DESs containing hydroxyl groups on HBDs exhibited higher NH_3_ absorption capacity than DESs containing amide groups. The optimal NH_3_ capacity of ChCl/EG (1:2) reached 46 mg g^−1^ at 303.15 K and 546.1 kPa. Moreover, NH_3_ absorption in this system was thermodynamically spontaneous according to thermodynamic property calculations, including standard Gibbs energy, dissolution enthalpy, and dissolution entropy [60]. Huang et al. [61, 62] further proposed introducing an HBD component with weak acidity into a ChCl-based system, which not only improved the NH_3_ absorption capacity but also resulted in the reversible absorption of NH_3_.

**Fig. 6:**
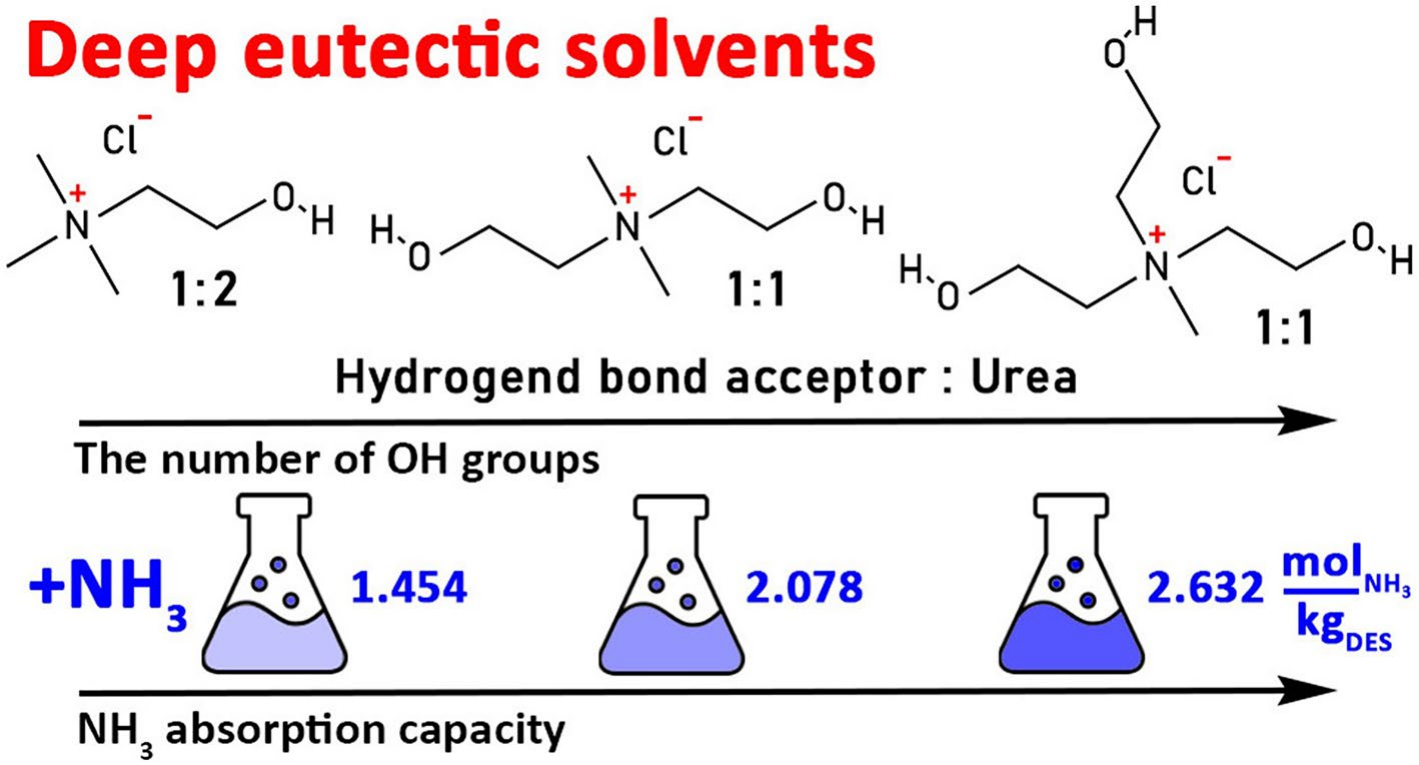
Structure of HBAs modified by hydroxyl groups on cation and NH_3_ absorption performance. Reproduced with permission from Ref. [66]. Copyright 2022, American Chemical Society

Considering the potential risk that toxic components in DESs pose to human health and the environment, Li et al. [63] proposed “natural DESs” composed of ChCl and sugar. These DESs exhibited higher NH_3_ capacities at low pressure and increased temperature compared with other reported DESs, which is important for practical applications, especially for low-concentration NH_3_ capture. Most studies have focused on the rational design of HBD structures in DESs to regulate the NH_3_ absorption performance, whereas Kazarina et al. [64–66] considered the functional group modification of HBAs. The substitution of hydroxyl groups in ChCl, as shown in Fig. 6, remarkably decreased the toxicity and enhanced NH_3_ solubility via hydrogen bond interactions. The NH_3_ absorption capacity was enhanced by increasing the number of hydroxyl groups of the choline cation. The absorption capacities of [Me_2_C^OH^_2_N]Cl/Urea with two hydroxyl groups and [MeC^OH^_3_N]Cl/Urea with three hydroxyl groups were 35.3 and 44.7 mg NH_3_/g DES at 313.2 K and 101.3 kPa, respectively, which is approximately twice that of ChCl/urea (2:3) under the same conditions.

In addition to ChCl-based DESs, much less expensive ethylamine hydrochloride (EaCl)-based DESs have also been developed. Similarly, the EaCl-based DESs with different hydrogen-bond donating ability or Brønsted acidity such as EaCl/AA [67], EaCl/Gly [68], EaCl/PhOH [69], and EaCl/Res [70], were explored for NH_3_ capture. The effects of EaCl/HBD molar ratio, temperature, and pressure were investigated systematically. An appropriate EaCl/HBD molar ratio is beneficial for obtaining DESs with low viscosity and high NH_3_ absorption capacity. Decreased temperature and increased pressure contributed to enhanced NH_3_ absorption capacity.

However, the above-mentioned Cl-containing DESs have potential corrosivity hazards toward equipment in the practical applications. Zheng et al. [71] further proposed non-chloride DESs with multiple weak acidic sites (one carboxylic group and two phenolic hydroxyl groups) by dihydroxybenzoic acid (DHBA) and EG for selective NH_3_ absorption. The DHAB/EGs DESs provided multiple hydrogen bond sites with NH_3_ molecules, enabling exceptional and reversible NH_3_ absorption with the value of 199 mg g^−1^ at 100 kPa and 298.15 K. Additionally, imidazole-based ILs without chloride elements were prepared as DESs, such as [Bmim][MeSO_3_]/urea [72], and [Im][NO_3_]/EG [73]; protic imidazole IL-based [Im][NO_3_]/EG DES with a molar ratio of 1:3 exhibited the highest capacity of 211 mg NH_3_/g DES at 303 K and 100 kPa and great NH_3_/CO_2_ selectivity of 139.6 along with good recyclability. In addition, non-ILs binary DES systems have been developed for NH_3_ absorption, such as N-methylacetamide(MAA)/tetrazole [74], NH_4_SCN/Gly [75], and GI/AT [76], in which the optimal NH_3_ mass absorption capacity of NH_4_SCN/Gly (2:3) was as high as 223 mg/g DES at 303 K and 100 kPa because of the cooperative hydrogen bond interactions between NH_4_^+^, OH, and NH_3_ molecules.

## Porous Solids for NH_3_ Adsorption

**Fig. 7:**
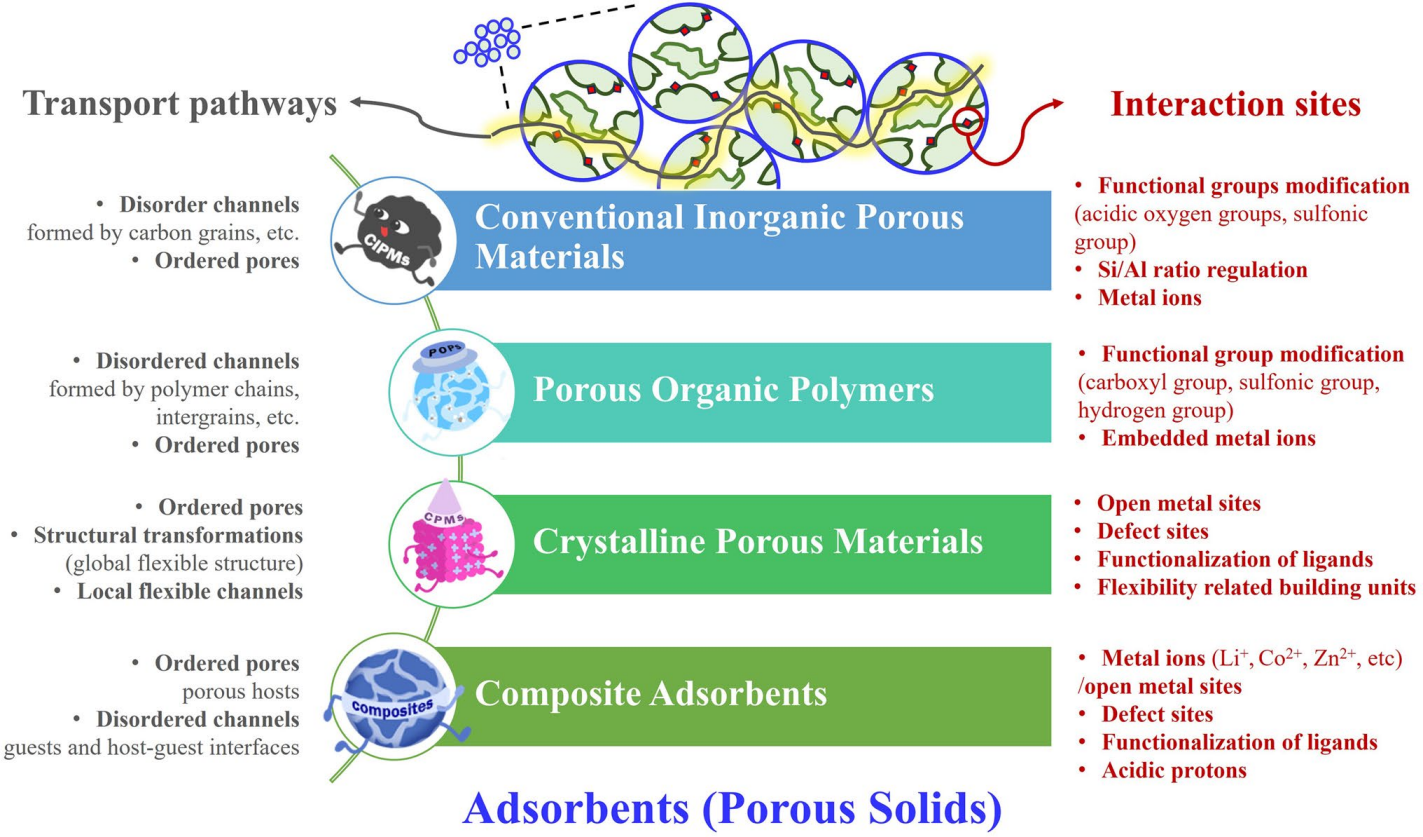
Interaction sites and transport ways of four types of porous solids

Compared with liquid absorption materials, porous solids for NH_3_ adsorption have been extensively studied [17, 77]. The abundant pores in solid materials provide the space for fast NH_3_ transport, which also avoids the problems of corrosion and low mass transfer efficiency resulting from acid scrubbing and the high viscosity of ILs. The reported porous solids can be roughly divided into four types: CIPMs, POPs, CPMs, and composite adsorbents, as shown in Fig. 7. Generally, CIPMs, such as activated carbon (AC), are low-cost and easy to fabricate, but the interaction between these materials and NH_3_ molecules is weak. As a potential solution, POPs have been exploited, by the disordered pores formed by polymer segments/ordered pores and acidic groups enhance the NH_3_ adsorption capacity. In addition, CPMs, such as MOFs, hydrogen-bonded organic frameworks (HOFs), and covalent organic frameworks (COFs, also belonging to POPs), usually exhibit high NH_3_ adsorption capacities and fast adsorption kinetics owing to their ordered pore structures and strong interactions with NH_3_. Furthermore, composite adsorbents, especially IL-based composites, which couple the high NH_3_ affinity of task-specific ILs with the porous properties of solid supports, are employed for NH_3_ capture. In this section, we focus on the novel adsorbents reported in the last 5 years, covering the design ideas of the corresponding materials, NH_3_ adsorption performance, and adsorption mechanism.

### Conventional Inorganic Porous Materials

Conventional inorganic porous materials (CIPMs), including activated carbons (ACs) [78–80], zeolite [77], metal halides [81], and mesoporous silica/alumina [82], have been widely used for NH_3_ capture because of their favorable characteristics, such as diverse pore architectures, high stability, and low cost. However, the NH_3_ adsorption capacity of these materials is relatively low owing to their limited affinity toward NH_3_ molecules. Therefore, the modification of functional groups to these CIPMs has been proposed to improve the NH_3_ adsorption capacity. For instance, Zheng et al. [83] developed fiber-form AC modified by acidic oxygen groups, which exhibited a high NH_3_ adsorption capacity of 50 mg g^−1^. Li et al. [84] found that AC modified by HNO_3_ exhibited the best NH_3_ removal performance among three inorganic acid-modified ACs, with a maximum NH_3_ adsorption amount of 40 mg g^−1^, owing to the reduced adsorption energy caused by the co-adsorption of NH_3_ with residual HNO_3_ via a hydrogen bond network. More recently, Zhang et al. [85] demonstrated that ordered MS functionalized with a sulfonic group (OMS-SO_3_H) exhibited ultra-high precision for NH_3_ reversible adsorption and separation, benefiting from the high density of –SO_3_H superic acid sites in ordered mesochannels.

Zeolites are another popular material for NH_3_ adsorption, and its properties, including acidity/basicity and hydrophilicity/hydrophobicity, can be tuned by varying the Si/Al ratio. Therefore, through interaction between NH_3_ and zeolites, NH_3_ adsorption performance can be finely regulated. Martos et al. [86] revealed that hydrogen bonds play an important role in NH_3_ capture with pure or high-silica zeolites, as confirmed by experimental and molecular simulations. Ouyang et al. [87] further indicated that NH_3_ adsorption capacity was inversely proportional to the Si/Al ratio. Exchanging the counter cation from Na^+^ to Li^+^ led to a higher NH_3_ adsorption capacity owing to stronger interactions between Li^+^ and NH_3_. In addition, the Al distribution in the nanopores and synthetic materials also affected the NH_3_ adsorption performance [88, 89].

Metal halides can effectively capture NH_3_ molecules by forming metal–ammonia complexes; however, regeneration at low temperatures is difficult [81]. Recently, Shen et al. [90] further studied the effect of metal halide types with the same metal cation and number of cycles on NH_3_ adsorption capacity. The NH_3_ adsorption capacity followed the order CuCl_2_ > CuBr_2_ > CuI, and these materials underwent severe sintering during the high-temperature regeneration process, causing difficulty in recycling. Cao et al. [91] exploited a novel porous SrCl_2_ structure with 96 wt% loading scaffolded by reduced graphene oxide networks to avoid sintering, which showed superior NH_3_ adsorption capacity (50.5 mmol g^−1^) and rapid absorption–desorption kinetics and maintained a porous structure accommodating the volume without disintegration during cycling experiments.

### Porous Organic Polymers

Porous organic polymers (POPs) are among the most widely studied materials for gas separation owing to their various monomer geometries and excellent thermal/chemical stabilities derived from the covalent nature of polymers [92, 93]. The functionalization of POPs with tunable and strong acid sites is an effective way to improve NH_3_ adsorption performance. In 2014, Van Humbeck et al. [94] firstly presented a series of diamondoid POPs densely functionalized with carbonylic acids for NH_3_ capture. Among various polymers, BPP-5 with a multiply interpenetrated structure dominated by pores smaller than 6 Å pores exhibits an NH_3_ uptake of 17.7 mmol g^−1^ at 1 bar, and BPP-7 with larger pore size shows improved NH_3_ absorption kinetics at low pressure (3.15 mmol g^−1^ at 480 ppm), but the recyclability of these POPs is not clear. Since then, various POPs modified by acid groups have been developed for NH_3_ adsorption, such as COOH-copolymer PDAB-AA [95], PIM-1-COOH [96], H_3_PO_4_ modified POPs [97], H_2_SO_4_-modified ethylene glycol dimethacrylate (EGDMA) polymers [98, 99] and sulfonated POPs [100], and the regeneration abilities of most materials have also been investigated systematically. Table 3 presents the NH_3_ adsorption performance and regeneration properties of representative POPs.

**Table 3:** NH_3_ adsorption capacity and regeneration properties of representative POPs

Porous organic polymers	Functional groups	NH_3_ adsorption capacity	Regeneration conditions	Adsorption loss	References
BPP-5	–COOH	17.7 mmol g^−1^ at 1 bar	NA^a^		[94]
BPP-7		3.15 mmol g^−1^ at 480 ppm			
PDVB-2.0AA	–COOH	3.53 mmol g^−1^ at 25 °C and 0.05 bar	80 °C, vacuum	11.2% (10 cycles)	[95]
PIM-1-COOH	–COOH	12.2 mmol g^−1^ at 1bar and 25 °C	RT and vacuum	~ 11.5% (3 cycles)	[96]
P2-CO2H	–COOH	16.1 mmol g^−1^ at 1bar and 25 °C	NA^a^		[98]
		3.15 mmol g^−1^ at 0.5mbar and 25 °C			
1-H_2_SO_4_-EGDMA	–COOH	5.06 mmol g^−1^ at 556 ppm and 20 °C	H_2_SO_4_ washing, vacuum 80 °C	No loss (4 cycles)	[99]
	–SO_3_H				
	–OH				
MPOP-1.0-SO_3_H	–SO_3_H	10.96 mmol g^−1^ at 1bar and 25 °C	Ar flow, 160 °C	No loss (4 cycles)	[100]
SOMPs	–HSO_3_	15.09 mmol g^−1^ at 25 °C and 1 bar	0.001 bar, 150 °C	1.6% loss (30 cycles)	[101]
		6.16 mmol g^−1^ at 25 °C and 0.05 bar			
1TCS	–COOH	2.94 mmol g^−1^ under humid conditions	NA^a^	No loss (10 cycles)	[102]
1TCS@PDMSX	–HSO_3_	2.1 mmol g^−1^ under humid conditions		No loss (4 cycles)	
1S	–HSO_3_	4.03 mmol g^−1^ at 500 ppm	NA^a^		[103]
1ESC9	–OH	0.74 mmol g^−1^ at 80% RH	He sweep	No loss (5 cycles)	
PAA	–COOH	10.7 mmol g^−1^ at 25 °C and 1 bar	80 °C, 4 h	35% loss (1 cycle)	[105]
	–NH–	1.6 mmol g^−1^ at 25 °C and 1mbar			
Ph2Im-TBB-TA	Co(SCN)_4_^2−^	20.1 mmol g^−1^ at 25 °C and 1 bar	80 °C, vacuum	No loss (5 cycles)	[106]
	C2H in im ring	5.2 mmol g^−1^ at 25 °C and 0.02 bar			
PVIm-R8-Co	Co^2+^	20.1 mmol g^−1^ at 25 °C and 1 bar	80 °C, vacuum	No loss (6 cycles)	[107]
Cu@PIP-X	Cu^2+^	10.2 mmol g^−1^ at 25 °C and 1 bar	100 °C, vacuum	No loss (5 cycles)	[93]
	B^−^				

^a^NA is the abbreviation of not available

**Fig. 8:**
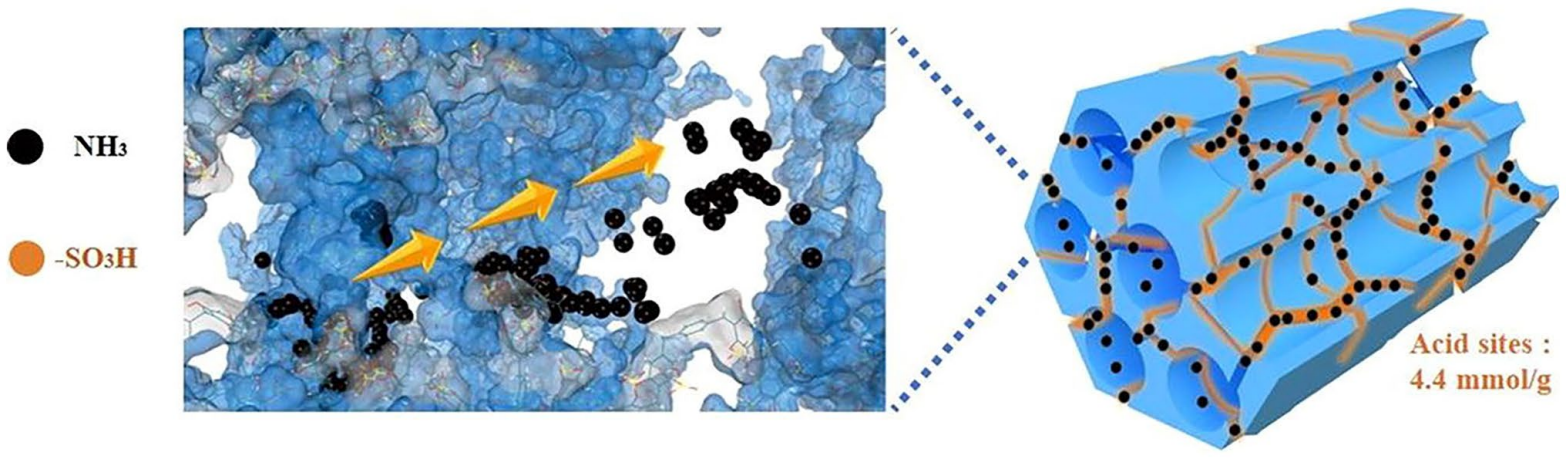
NH_3_ adsorption mechanism of SOMPs. Reproduced with permission from Ref. [101]. Copyright 2022, Elsevier

Recently, Kan et al. [101] further reported a sulfonated and ordered mesoporous polymer (SOMP). The strong affinity of the –HSO_3_ group with NH_3_ in sequential pore space of SOMPs, as shown in Fig. 8, not only enhances the molecular recognition ability but also facilitates NH_3_ fast diffusion inside SOMPs so that favorable NH_3_ adsorption capacity (15.09 mmol g^−1^ at 25 °C and 1 bar) and excellent reversibility could be achieved. Additionally, a multi-step post-modification strategy was proposed to further improve the NH_3_ adsorption performance of POPs. Kang et al. [102] found that a high NH_3_ adsorption capacity, especially at low pressures, and excellent recyclability were obtained owing to the formation of high-density acidic functional groups (–COOH and –HSO_3_) induced by post-oxidation and post-sulfonation processes on poly(dimethylsiloxane) (PDMS)-coated hyper-crosslinked POPs. Furthermore, sequential post-sulfonation and post-alkylation reactions were developed to modify POPs for NH_3_ capture. A record-high NH_3_ capacity (4.03 mmol g^−1^) at 500 ppm was achieved, and it adsorbed 0.48 mmol g^−1^ even at a concentration of 800 ppb. Simultaneously, the hydrophobic nature of alkyl chains offers rapid desorption kinetics and exceptional recyclability under dry and humid conditions at room temperature [103].

In addition to their ability to interact with acidic groups, the hydrogen bond-forming properties of NH_3_ molecules are highly attractive for POPs. Lima et al. [104] demonstrated that hydrogen bonds play an important role in the NH_3_ uptake by poly(amic acid) (PAA) by combining TGA with neutron spectroscopy, supported by DFT calculations. Besides, the hydrogen bond sites with –COOH groups efficiently improved NH_3_ adsorption performance of PAA, but strong interaction also made complete regeneration difficult [105].

**Fig. 9:**
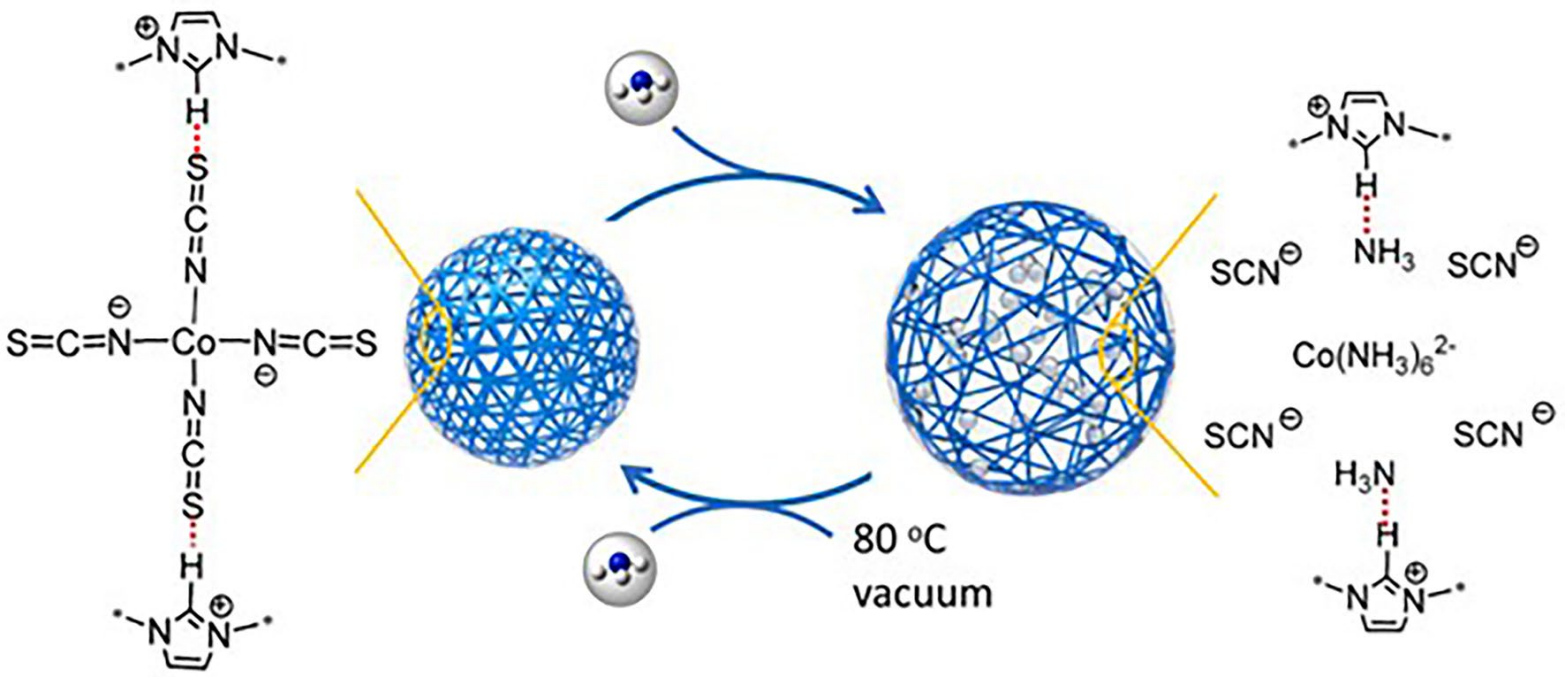
Proposed mechanism of NH_3_ adsorption with PIL-Tas. Reproduced with permission from Ref. [106]. Copyright 2021, Elsevier

The incorporation of open metal sites and ionic units to prepare porous ionic polymers as NH_3_ adsorbents is also promising. As shown in Fig. 9, Luo et al. [106] reported porous cobaltous thiocyanate (Co(II)(SCN)_4_^2−^, TA)-functionalized polyILs with an NH_3_ uptake capacity of 12.2–20.1 mmol g^−1^ owing to cooperative interactions containing NH_3_ coordinating with Co(II) instead of SCN^−^ and hydrogen bonding of H at the C2 atom in the imidazolium ring (C2H···NH_3_). At the same time, the competitive interaction between NH_3_ and free SCN^−^ promoted NH_3_ release, contributing to the good recyclability of the adsorbents. Moreover, the coordinative numbers of metal centers in polyILs with NH_3_ molecules have a significant effect on the NH_3_ capacity. For instance, high NH_3_ adsorption capacity was achieved when the coordination number increased from *n* = 4 (M = Cu/Zn) to *n* = 6 (M = Co). Increasing the moderate size of the cross-linking agent R enhances the NH_3_ capacity of PILs; however, oversizing R also reduces the porosity of polyILs [107]. Similarly, PIPs have also been explored, and multiple active sites containing charged skeletons, Lewis acid defects, and metal ions in Cu@PIP jointly promoted improved NH_3_ adsorption performance and outstanding recyclability without structural collapse [93].

### Crystalline Porous Materials

MOFs, which are typical CPMs, are one of the most promising candidates for NH_3_ adsorption because their sorption selectivity is directly tunable as a function of the topology and chemical composition of the pores [19, 108–112]. Takahashi et al. [113] firstly demonstrated the possibility of historical pigment of Prussian blue and its analogs (CoHCC and CuHCF) for NH_3_ capture. The NH_3_ uptake capacity of Prussian blue is up to 12.5 mmol g^−1^ at 0.1 MPa owing to the NH_3_ transformation into NH_4_^+^ with H_2_O in air, which is much higher than that of standard NH_3_ adsorbents (5.08–11.3 mmol g^−1^). Subsequently, various MOFs for efficient NH_3_ adsorption have been developed, in which the incorporation of open metal sites, functional sites on the ligand, and defect sites are effective measures to improve NH_3_ adsorption performance. The NH_3_ adsorption performance and regeneration properties of representative MOFs are listed in Table 4.

**Table 4:** NH_3_ adsorption capacity and regeneration properties of representative CPMs

crystalline porous materials	T (°C)	P (bar)	NH_3_ adsorption capacity (mmol g^−1^)	Regeneration conditions	Adsorption loss	References
CoHCC	25	1	21.9	Vacuum,150 °C	No loss (4 cycle)	[113]
Co_2_Cl_2_(BBTA)	25	1	17.95	Vacuum, 200 °C	~ 4% (3 cycle)	[114]
Rh-MOPs	25	1	12.9	Vacuum, 130 °C	52% (1 cycle)	[115]
CuBTC	25	1	23.88	Structural collapse		[116]
	25	BC^a^	8.8			[126]
Zr_6_-NU-907	25	1	12.1	31.4% (3 successive cycles)		[118]
Al-PMOF	25	1	7.67	No loss (2 successive cycles)		[117]
Mg_2_(dobpdc)	25	1	23.9	No loss (5 consecutive cycles)		[119]
	25	BC^a^	8.37			
SION105-Eu	30	1	5.7	70 °C for 30 min	No loss (5 cycles)	[120]
MFM-303(Al)	25	BC^a^	2.9	Vacuum, 80 °C	17% loss (29 cycles)	[121]
UiO-66-Cu^II^	0	1	16.9	Vacuum	No loss (15 cycles)	[125]
	25	BC^a^	4.15			
MFM-300(Al)	25	1	13.9	Dynamic vacuum < 1 h	No loss (50 cycles)	[129]
CAU-10-OH	25	BC^a^	3.5	Vacuum, 80 °C for 6 h	No loss (6 cycles)	[131]
Zn(NA)_2_	25	1	10.2	Vacuum, 150 °C for 70 min	No loss (3 cycles)	[134]
KUF-1^a^	25	1	6.67	Vacuum, RT^b^	No loss (5 cycles)	[135]
[SrOOC]17-COF	10	1	19.8		44.8% loss (3 consecutive cycles)	[140]

^a^BC is the abbreviation of breakthrough capacity

^b^RT is the abbreviation of room temperature

**Fig. 10:**
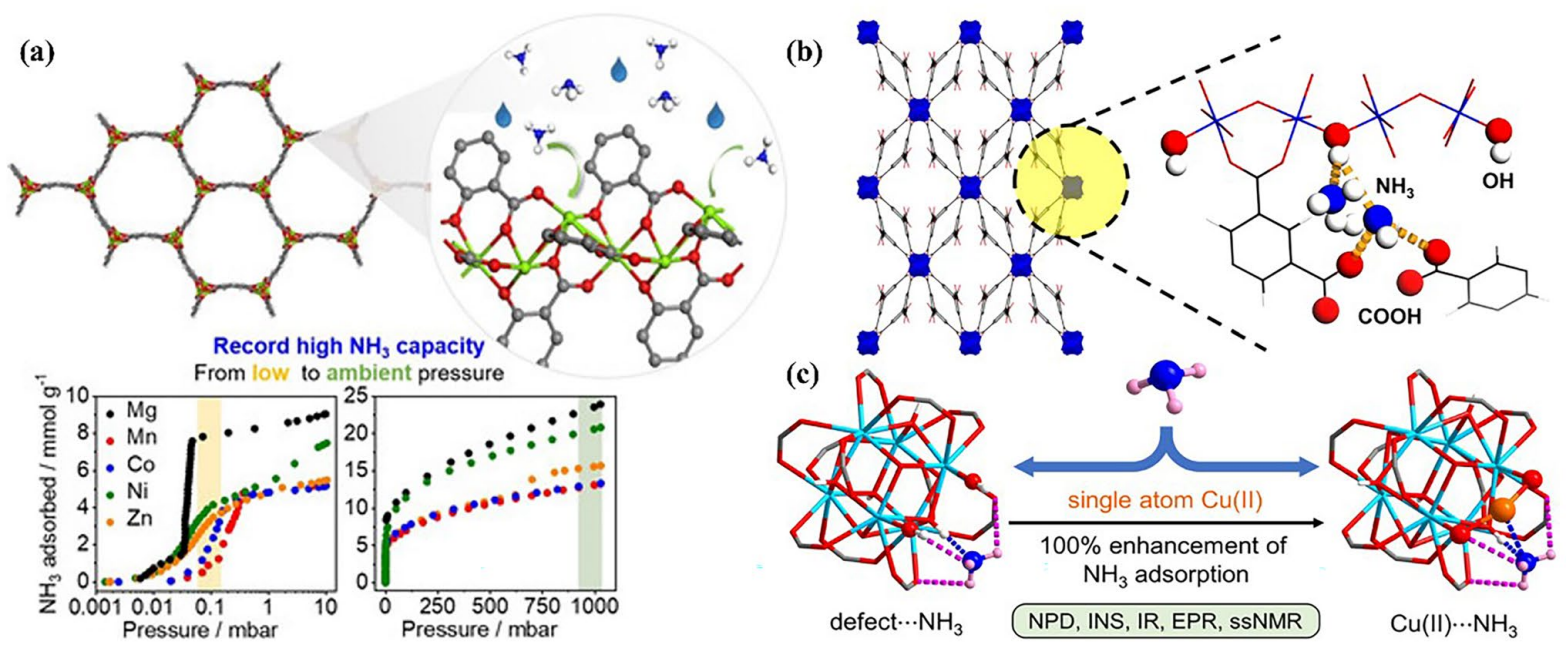
**a** Atomic structure of M_2_(dobpdc) (M^2+^ = Mg^2+^, Mn^2+^, Co^2+^, Ni^2+^,and Zn^2+^) and NH_3_ adsorption isotherms at 298 K. Reproduced with permission from Ref. [119]. Copyright 2020, John Wiley and Sons. **b** Structure of MFM-303(Al). Reproduced with permission from Ref. [121]. Copyright 2021, American Chemical Society. **c** Structure of UiO-66 material with defect site and Cu(II) Reproduced with permission from Ref. [125]. Copyright 2022, American Chemical Society

The incorporation of open metal sites in MOFs provides high NH_3_ affinity, which is expected to remarkably improve NH_3_ adsorption performance. For example, Dinca’s group developed a series of triazole MOFs with open metal sites (Co, Ni, and Cu), in which Co_2_Cl_2_BBTA (BBTA = 1*H*,5*H*-benzo(1,2d), (4,5-d′)bistriazole) with smaller-pores exhibited greater capacities than their larger-pores BTDD-based counterparts (BTDD = bis(1*H*-1,2,3triazolo[4,5-b],[4′,5′-i])dibenzo[1,4]dioxin) benefiting from the higher density of open metal sites. The static uptake is up to 19.79 mmol NH_3_ g^−1^ at 1 bar and 298 K, which is more than twice the capacity of commercial activated carbon [114]. In contrast to the above-mentioned formation of NH_3_-binding sites triggered by high-quality clusters, Carne-Sanchez et al. [115] reported metal–organic polyhedrals (MOPs) with Rh(II) as open metal sites for NH_3_ capture. The low nuclear Rh(II) paddlewheel clusters in the synthesized MOFs firstly coordinated with NH_3_ molecules, which further induced the adsorption of additional NH_3_ molecules through H-bond interaction. This unique mechanism endows the prepared Rh-MOPs with a high NH_3_ adsorption capacity exceeding 10 mmol g^−1^, which can be easily regenerated via immersion in an acidic solution. The effect of open metal sites on NH_3_ adsorption behavior was also investigated systemically. For example, CuBTC exhibited a higher ammonia uptake of 23.88 mmol g^−1^ compared with others (ZnBTC, 11.33 mmol g^−1^; FeBTC, 9.5 mmol g^−1^) [116], and Al-PMOF showed greater NH_3_ adsorption reversibility compared to those of isoreticular Ga-PMOF and In-PMOF [117]. Likewise, Zr_6_-NU-907 exhibited the highest NH_3_ adsorption capacity at low pressure among the NU-907 family of MOFs, owing to the higher electronegativity of metal Zr ions. The in situ IR results further demonstrated that NH_4_^+^ and Lewis-bound NH_3_ species were formed during NH_3_ adsorption [118]. The Mg_2_(dodpdc) exhibited record-high NH_3_ capacity of 23.90 mmol g^−1^ at 1 bar and 8.25 mmol g^−1^ at a low pressure of 0.57 mbar in a series of M_2_(dodpdc) MOFs that were constructed from various divalent metal cations (*M* = Mg^2+^, Mn^2+^, Co^2+^, Ni^2+^, and Zn^2+^) and a tetradentate ligand dobpdc^4−^ (Fig. 10a). At the same time, it was confirmed that Mg_2_(dodpdc) shows excellent structural stability even for wet NH_3_ owing to the higher affinity of Mg^2+^ for oxygen atoms than for nitrogen atoms, showing great potential for practical applications [119].

Functionalization of the ligand is another effective strategy for improving the NH_3_ adsorption performance of MOFs. Nguyen et al. [120] functionalized ligands by incorporating electrophilic boron (B) centers, which electrophilized the pores and promoted the capture of electrostatic NH_3_ molecules. Moreover, the bulky duryl groups precluded strong acid–base B-N interactions to ensure the robustness of MOFs in the presence of NH_3_. Therefore, the prepared SION105-Eu MOF via this strategy is easily regenerated after NH_3_ adsorption by simple heating at 75 °C. In addition, a robust Al-based MOF, MFM-303(Al), functionalized with free carboxylic acid and hydroxyl groups, was developed for NH_3_ capture, as shown in Fig. 10b [121]. Two carboxylate groups from each tetracarboxylic ligand molecule were bound to the Al(III) centers, whereas the other two remained uncoordinated and formed intramolecular hydrogen bonds with neighboring ligands. These acidic sites in the pores not only make MFM-303(Al) show excellent adsorption performance for low concentrations of NH_3_ under both dry and wet conditions but also offer an exceptional packing density of NH_3_ at 293 K (0.801 g cm^−3^), comparable to that of solid NH_3_ at 193 K (0.817 g cm^−3^), which means that MFM-303(Al) could be used for NH_3_ storage in practical applications.

Defective MOF construction by missing ligands or metal nodes can tune the nanostructure and pore size [122–124], thereby affecting the NH_3_ adsorption performance. Ma et al. [125] simultaneously introduced defect sites and open Cu(I) and Cu(II) sites on a robust UiO-66 material, as shown in Fig. 10c, which exhibited high and reversible NH_3_ adsorption capacity, avoiding the issues that MOFs with multiple coordination Cu(II) sites suffer from irreversible NH_3_ sorption and structural degradation during adsorption. The excellent NH_3_ adsorption–desorption reversibility of these MOFs predominantly resulted from the reversible change in the near-linear coordination geometry of the Cu(II) sites as a function of NH_3_ binding.

Although MOFs possess high adsorption capacity and selectivity, most of them still face the challenge of structural degradation when in contact with NH_3_ [126, 127]. Thus, enhancing the strength of the coordination bonds and tuning the moderate interaction between metal centers and NH_3_ when designing MOFs should be emphatically considered. Yang et al. [128] designed a kind of ultra-stable MFM-300(Al), in which AlO_4_(OH)_2_ was bridged by 3,3′,5,5′-bipphenyl-tetracarboxylic acid to form a “wine-rack” framework, which could store ammonia for at least 183 weeks without decreasing in the apparent domain size of changes in the unit cell volumes. The NH_3_ adsorption capacity reached 15.7 mmol g^−1^ at 273 K and 1.0 bar, and there was no loss of NH_3_ adsorption capacity over 50 cycles owing to the reversible H/D site exchange between the MOF and NH_3_ molecules revealed by in situ neutron powder diffraction and synchrotron FTIR micro-spectroscopy [129]. O Other isostructural analogs of MFM-300(M) (M = Fe, Cr, V) have also been reported, among which MFM-300(M) (M = Cr, V^III^) showed higher stability against humid NH_3_ than MFM-300(M) (M = Fe, V^IV^) [130]. Wang et al. [131] further synthesized super-stable CAU-10-based MOFs by choosing relatively inert Al^3+^ as metal nodes. Besides excellent long-term stability (more than 2 years), hydroxyl-functionalized CAU-10-O showed ultrahigh NH_3_ adsorption capacity (3.5 mmol g^−1^ at 25.0 °C) for low-concentration NH_3_ (5000 ppm), high selectivity of NH_3_ to N_2_ (up to 4850), and mild regeneration conditions (80 °C under vacuum for 6 h). Such great separation performance of CAU-10-OH was attributed to the multiple hydrogen bonding interactions (μ-OH···NH_3_ and –OH···NH_3_) between NH_3_ and CAU-10-OH.

**Fig. 11:**
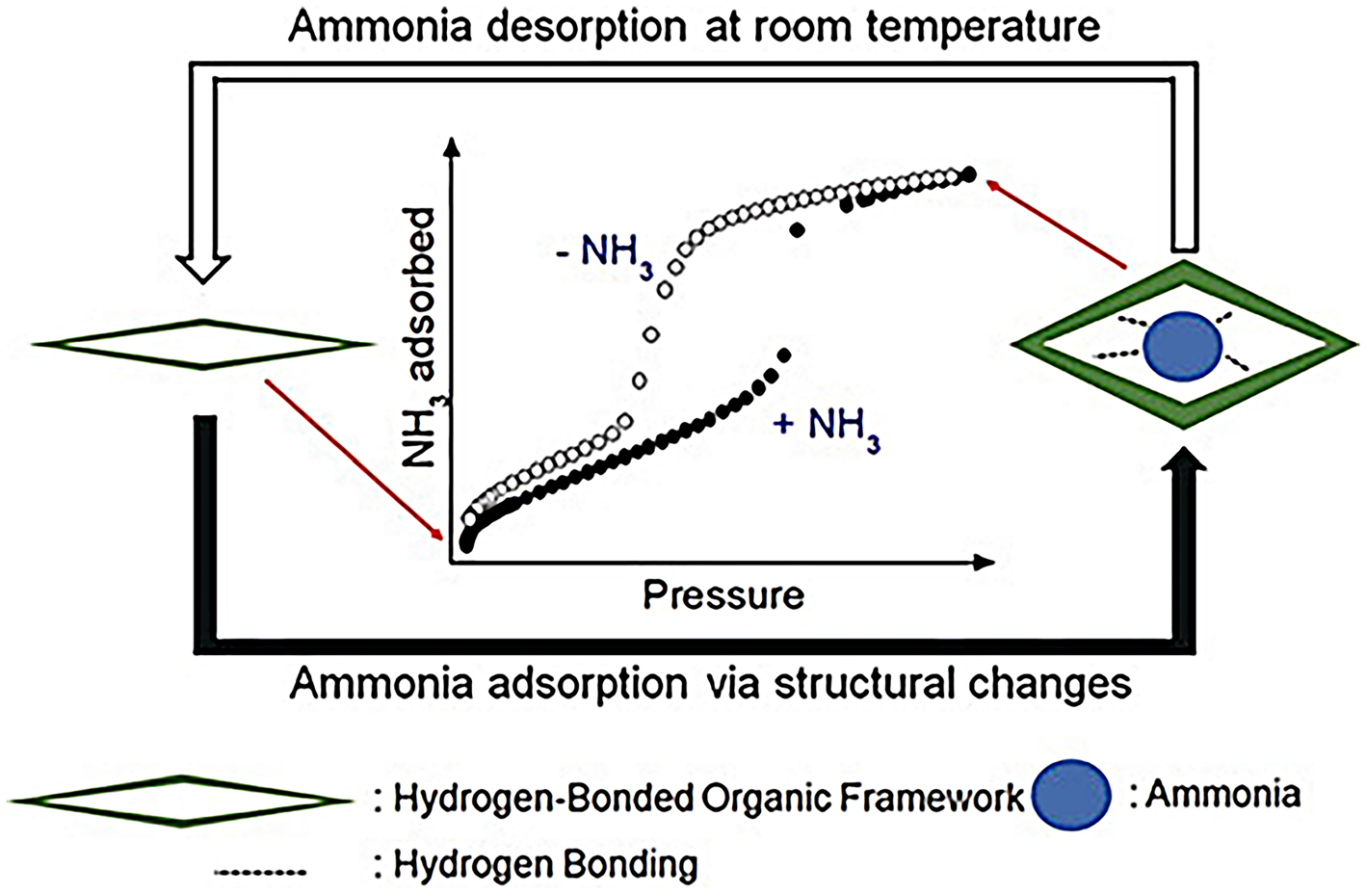
Type IV NH_3_ adsorption isotherm by HOF KUF-1a (Reproduced with permission from Ref. [135]. Copyright 2020, John Wiley and Sons

Flexible MOFs with reversible structural transformations (topology, pore size or shape) usually exhibit a steep S-shaped adsorption curve, which can realize a high NH_3_ working capacity and facile regeneration [132, 133]. For example, Chen et al. [134] found that the dehydration of M(NA)_2_(H_2_O)_4_, (M = Zn, Co, Cu, Cd, NA = nicotinate) induces a structural transformation from zero-dimensional (0D) to two/three-dimensional (2D/3D), which is reversible after water adsorption. The layered 2D Zn(NA)_2_ exhibited a gate-opening effect at a pressure of 0.22 bar, leading to a two-step NH_3_ uptake with a capacity of 10.2 mmol g^−1^ at 1 bar, while 2D Co(NA)_2_ showed a continually increasing NH_3_ trend with an increase in pressure and an NH_3_ adsorption capacity up to 17.5 mmol g^−1^. For the transformed 3D Cu(NA)_2_ and Cd(NA)_2_, higher NH_3_ adsorption rates and shorter adsorption equilibrium times were achieved after three cycles. Meanwhile, both MOFs showed great recyclability and could be regenerated under vacuum and heating conditions of 150 °C for 70 min. Similarly, Kang et al. [135] developed a novel sorbent called flexible HOFs KUF-1 for NH_3_ adsorption, which showed unprecedented type IV adsorption behavior in the NH_3_ isotherm at 298 K, with a capacity of 6.67 mmol g^−1^ at 1 bar (Fig. 11). This material can be completely regenerated at room temperature under vacuum. In addition, FDU-HOF-3 with self-healing properties and excellent capture performance at low NH_3_ (8.13 mmol g^−1^ at 25 mbar) has also been developed [136]. Encouraged by reversible hydrogen network, Li et al. [137] further developed ionic frameworks [Ph3ImH][NTf_2_]_2_ constructed from protic imidazolium ILs units through ionic and hydrogen bonding interactions for NH_3_ capture, which presented selective NH_3_ capture of 15.65 mmol g^−1^ (25 °C and 1 bar) and mild regeneration conditions (80 °C and 1 mbar).

**Fig. 12:**
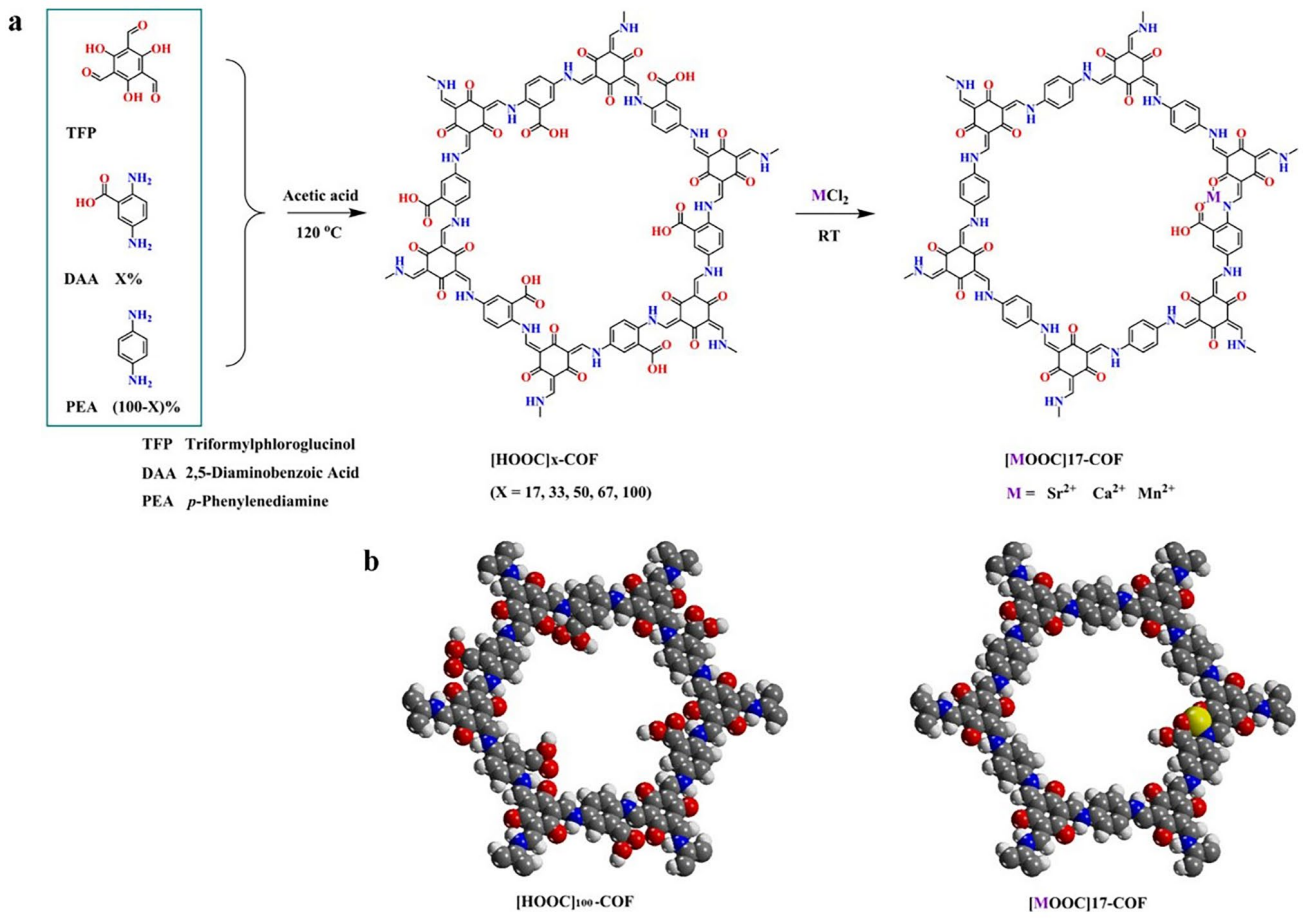
**a** Scheme for surface pore engineering of COFs with various groups.** b** Possible pore structure of COFs with various groups (gray, C; blue, N; red, O; yellow, metal) (Reproduced with permission from Ref. [140]. Copyright 2018, American Chemical Society

Another emerging CPM, covalent organic frameworks (COFs), have also been explored for NH_3_ adsorption. In contrast to MOFs, COFs are formed by connecting light atoms (hydrogen, boron, carbon, nitrogen, etc.) via strong covalent bonds. Thus, COFs usually exhibit higher NH_3_ stability than MOFs based on the difference in bond strength. In addition, they have ordered pore structures that can effectively adsorb NH_3_ molecules and be regulated according to the specific separation conditions [138, 139]. Inspired by the functional design of MOFs, decorating the pore walls of COFs with various open metal sites has also been proposed to improve NH_3_ adsorption performance. For example, Yang et al. [140] adopted a surface pore engineering strategy to design multivariate COFs by decorating the pore walls with various functional units for NH_3_ adsorption (Fig. 12). Owing to the high NH_3_ affinity of synergistic multivariate and open metal sites, the COFs exhibited high NH_3_ adsorption capacities (14.3 and 19.8 mmol g^−1^ at 298 and 283 K, respectively). Zhao et al. [141] investigated the NH_3_ adsorption properties of COF-10 and its Li-doped derivatives using simulations. The NH_3_ adsorption capacity could be improved by introducing more charged lithium atoms; however, this was not proportional to the number of lithium atoms. In addition, the charge distribution also affected the NH_3_ adsorption behavior. In particular, a positive potential shield on the surface of COF-10-6Li protected NH_3_ from negative charge repulsion on the inner skeleton; thus, a remarkable enhancement in the NH_3_ adsorption capacity was observed when six lithium atoms were introduced.

The actual performances of various materials under working conditions (containing H_2_O and impurity molecules in handling gases) are important for practical applications. Although several MOFs have been explored, the number of MOFs reported for NH_3_ capture from complex environments remains limited. Liu et al. [142] conducted a high-throughput computational screening (HTCS) of 2932 CoRe MOFs based on grand canonical Monte Carlo (GCMC) simulations to screen for the optimal MOF for NH_3_ capture from humid gas. They found that the key to a high NH_3_ capture performance was affinity or Henry’s constant of MOFs toward NH_3_ and water molecules. Previous research has indicated that NH_3_ uptake by MOFs mostly exhibits a solubilization-like mechanism in the presence of H_2_O molecules [143]. Hydrophobic MOFs possessed higher NH_3_ selectivity, while hydrophilic MOFs exhibited higher NH_3_ uptake despite strong adsorption competition from H_2_O molecules. In addition, the presence of H_2_O molecules could promote the enhancement of NH_3_ uptake in the MOFs with a coefficient (describing the effect of H_2_O adsorption on NH_3_ uptake) of IC_H2O_NH3_ < 0, but their ammonia uptake was still lower than that with IC_H2O_NH3_ > 0, which is important for the structural design of MOFs. In fact, in NH_3_-contaning gas from different NH_3_ emission sources, not only are H_2_O molecules present, but there are also other impurities such as SO_2,_ which also greatly affect the NH_3_ adsorption behaviors. Chen et al. [144] combined adsorption isotherms with DFT calculations to investigate this effect at low pressure. It was found that NH_3_ is the most affinitive molecule to HKUST-1 among three molecules, while SO_2_ was the most affinitive molecule to UIO-66; therefore, NH_3_ is likely to displace pre-adsorbed SO_2_ or H_2_O on HKUST-1. Also, there is chemical adsorption on HKUST-1 and MIL-100(Fe) toward NH_3_, while NH_3_ adsorption to UIO-66 likely involves physisorption.

### Composite Adsorbents

**Fig. 13:**
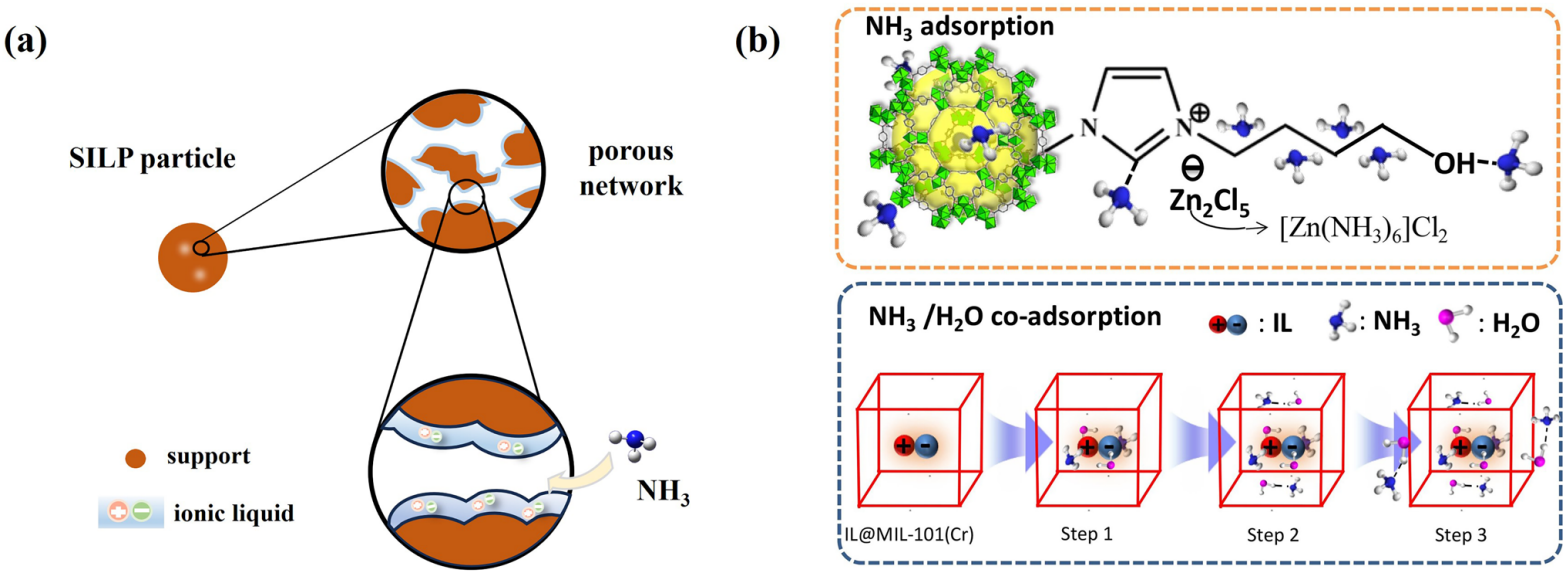
**a** Schematic diagram of supported ILs phase materials. **b** Schematic diagram of mechanism of [BOHmim][Zn_2_Cl_5_]@MIL-101(Cr). Reproduced with permission from Ref. [156]. Copyright 2020, Elsevier

Composite adsorbents combine the advantages of different materials in terms of NH_3_ adsorption, such as metal chloride/carbon cubes, metal chloride/COFs, and IL-based composites, showing good development prospects for NH_3_ capture [145–148]. As a representative composite material, supported IL-phase (SILP) materials (Fig. 13a) have received more attention due to the cooperative effect of the porous support and functional ILs [48, 149, 150], which also solve the problems associated with the application of highly viscous or solid ILs for NH_3_ separation. Functional ILs in SILP materials mainly provide high NH_3_ affinity via interaction sites (see Sect. 3.1). Porous supports not only provide NH_3_ transport pathways but also effectively disperse ILs to expose more accessible sites of ILs to further improve the NH_3_ adsorption capacity.

Pioneering work on SILP for NH_3_ adsorption was reported in 2014, where an AC support material was coated with [C_2_C1Im]Cl/CuCl_2_. The superior NH_3_ adsorption capacity was predominantly attributed to strong interactions between Cu^2+^ and NH_3_ molecules [48]. However, regeneration was difficult under mild conditions. Therefore, subsequent studies have focused on the design of SILP materials for reversible NH_3_ adsorption, in which the appropriate selection and matching of supports and ILs are of great importance. Yu et al. [151] selected protic ILs with reversible NH_3_ absorption to be supported on AC with low cost and large surface area for NH_3_ adsorption. The results showed that 20 wt% [2-Mim][NTf_2_]@AC-980 exhibited a higher NH_3_ adsorption capacity of 68.61 mg g^−1^ NH_3_ at 303.15 K and 0.10 MPa (30% higher than that of pure AC) and excellent recyclability, benefiting from the synergistic interaction of hydrogen bonding between the ILs and NH_3_ and hierarchical pores. To further improve the NH_3_ adsorption performance, various task-specific ILs with multiple hydrogen-bond interaction sites/complexation sites and porous supports with different pore sizes were utilized to develop a variety of SILP materials, such as multiphoton ILs@HZSM-5 [152], hydroxyl ammonium protic ILs@MCM-41 [153], Zn-based ILs@FDU-12 [154], and MILs [Bmim]_2_[Co(NCS)_4_]@silica composites [155]. Enhanced NH_3_ adsorption capacity and excellent adsorption–desorption performance were achieved in these materials, presenting great potential for application.

Encouraged by their tunable pore structure and chemical composition, MOFs have also been exploited as porous supports for constructing versatile SILPs. Han et al. [156] fabricated a highly stable IL@MOF composite material for NH_3_ capture for the first time. [BOHmim][Zn_2_Cl_5_]@MIL-101(Cr) exhibited superior NH_3_ uptake of 24.12 mmol g^−1^ at 298 K and 1 bar, and such high NH_3_ adsorption capacity could be maintained under humid NH_3_ conditions. This excellent performance was related to the synergistic effect of multiple adsorption sites and the large free transport space provided by alkyl chains. Moreover, a small amount of adsorbed water provided additional NH_3_ uptake, as shown in Fig. 11b. Subsequently, a [CAM][Cl]@MIL-101(Cr) composite was developed, and high-purity NH_3_ was obtained in one step, as proven by a breakthrough experiment of an NH_3_/CO_2_ mixture showing superhigh NH_3_/CO_2_ separation factor of up to 1518 [157]. Shi et al. [158] anchored 43.4 wt% LiCl into the nanopores of MIL-53-(OH)_2_ by charge transfer and hydrogen bonding for NH_3_ capture. A record NH_3_ adsorption capacity (33.9 mmol g^−1^ at 1.0 bar and 25 °C) and superior selectivity of NH_3_/N_2_ (3571 at 25 °C), NH_3_/CO_2_ (30.3 at 80 °C) and NH_3_/H_2_O (15.6 at 50 °C) were achieved owing to synergistic action of NH_3_ coordination with the highly dispersed Li^+^ in the MOF nanopores and hydrogen bonding of NH_3_ with Cl^−^.

**Fig. 14:**
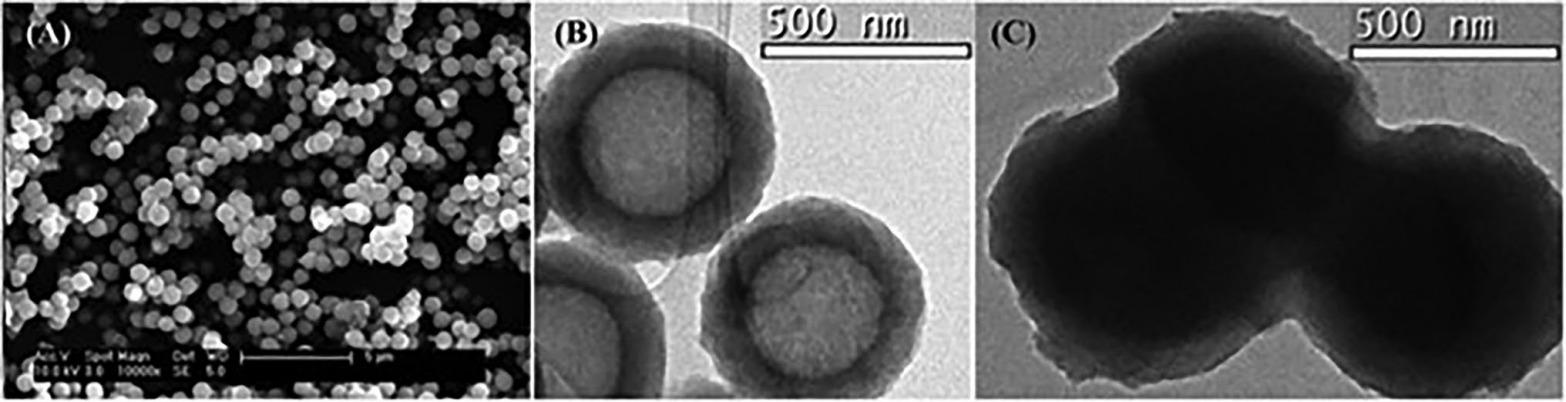
**a** SEM image,** b** TEM images of hollow carbonaceous submicrocapsules. **c** TEM image of ENIL prepared with [EtOHmim][BF_4_]. Reproduced with permission from Ref. [163], Copyright 2016, Royal Society of Chemistry

SILPs simultaneously improved the NH_3_ adsorption capacity and promoted NH_3_ transport, but the loading of ILs was always lower; therefore, the merits of liquid ILs were not fully displayed for the separation process [159–161]. A novel encapsulated ionic liquid (ENIL) was developed to achieve high IL loading and fully utilize the characteristics of ILs in confined spaces. Simultaneously, this material achieved the discretization of ILs from continuous to small drops, thereby increasing the surface contact area and improving the mass transfer rate. Palomar et al. [162, 163] prepared an ENIL by confining [EtOHmim][BF_4_] into hollow carbon submicron capsules. The unique core–shell structure as shown in Fig. 14 not only preserves the high NH_3_ affinity and fluidity properties of ILs but also accelerates the absorption–desorption process compared with the continuous ILs phase. High IL content (> 85 wt%), nearly identical sorption capacity to pure ILs, and excellent regeneration properties were achieved in ENILs, providing a pioneering strategy for designing novel IL composites with ultrahigh IL loading for efficient NH_3_ separation.

## Porous Liquids for NH_3_ Ab-Adsorption

**Fig. 15:**
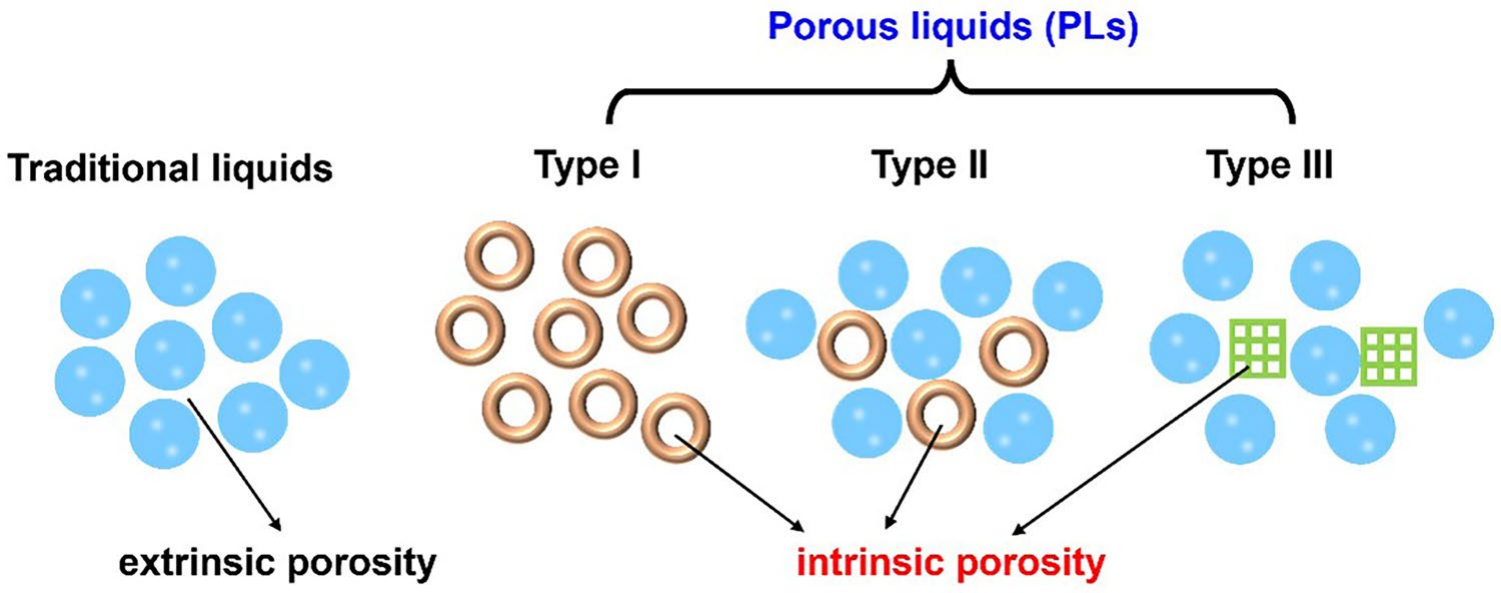
Schematic diagram of traditional liquids and three different types of PLs. Adapted from Ref. [164]

Porous liquids (PLs) are attractive materials that combine the permanent porosity of porous solids with liquid fluidity so that they can be easily coupled with existing process equipment, such as pumps and pipelines. Different from traditional liquids consisting of only random, transient cavities between the liquid molecules (here called “extrinsic” porosity), PLs are made of porous hosts possessing persistent empty cavities (called “intrinsic” porosity), which are able to work as a gas transport pathway to provide rapid adsorption and high capacity. The concept of PLs was first proposed by James and coworkers [164] and can be divided into three types according to the existing way of the porous hosts as shown in Fig. 15 [165–167]. Type I is a neat liquid composed of fluid hosts with empty cavities, whereas Type II and Type III are essentially dissolved empty hosts or homogeneously dispersed framework materials in sterically hindered solvents, respectively. To date, the application of PLs has focused on gas capture and storage [167–170], while the synthesis of stable PLs remains a significant challenge owing to intermolecular self-filling, collapse, or decomposition of the organic hosts and serious settling of solid particles.

**Fig. 16:**
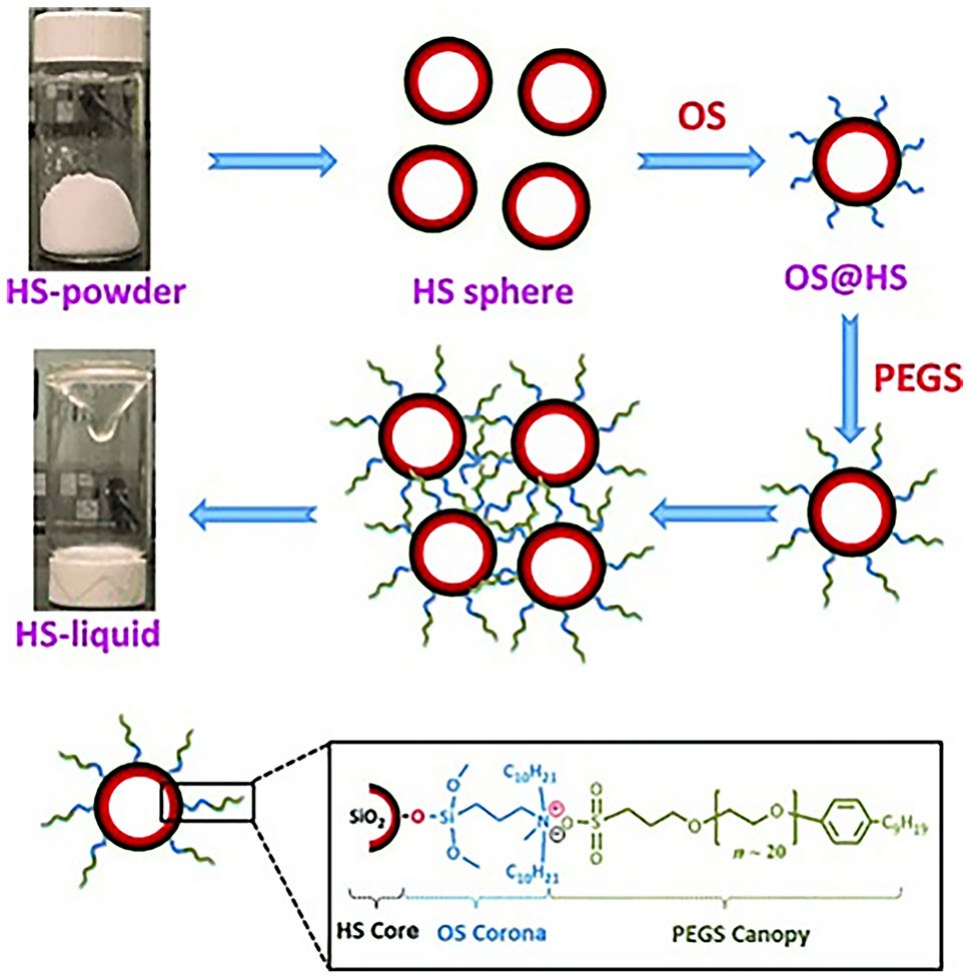
Two-step synthetic strategy for porous liquid fabrication. HS, hollow silica, OS, organosilane. Reproduced with permission from Ref. [167]. Copyright 2014, John Wiley and Sons

The synthesis of fluid hosts with empty cavities is the key to obtaining Type I PLs. Giri et al. [171] grafted medium-length alkyl tails onto the surfaces of rigid organic iminospherand cages to synthesize PLs. Alkylation obviously reduced the melting point of the cage from > 300 °C to as low as 50 °C, making it possible to obtain fluid cages with empty cavities at relatively low temperatures. The liquidation of reported porous materials is also an effective method for preparing PLs (PLs prepared by this method are also called Type IV PLs, in which porosity is offered by non-discrete molecular species [172]). For example, Gaillac et al. [173] studied the melting process and liquid nature of porous ZIF-4 using in situ variable temperature XRD, ex situ neutron pair distribution function (PDF), and first-principles molecular dynamics (FPMD). They verified that the porosity of ZIF-4 was retained after melting process. In addition, hollow carbon or silica spheres grafted with ILs is another facile strategy for preparing a new PL phase. Zhang et al. [167] grafted positively charged organsilane onto the surface of hollow silica spheres, followed by an anion exchange reaction to prepare HS-liquid at room temperature, as shown in Fig. 16. TEM images, N_2_-sorption isotherms, and small-angle X-ray scattering (SAXS) data revealed that well-defined hollow spheres were obtained. More importantly, the empty cavities significantly promoted CO_2_/N_2_ separation, showing attractive properties for target-specific applications, such as NH_3_ separation.

**Fig. 17:**
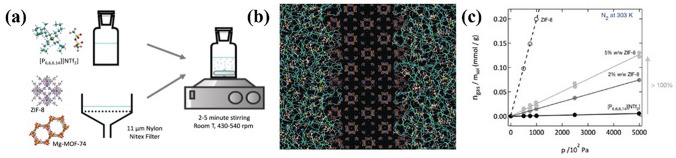
**a** Preparation of the porous liquids. **b** Molecular simulation of the porous liquids show empty pores in ZIF-8.** c** Dissolution of N_2_ in the PLs. Reproduced with permission from Ref. [27]. Copyright 2018, John Wiley and Sons

In addition, the various MOFs and functional solvents used for NH_3_ capture, as reported above, offer more opportunities for preparing Type-III PLs. The combination of MOFs with specific sterically hindered solvents, such as ILs, is expected to form new PLs with high gas uptake and separation performance. For example, Type-III PLs, including ZIF-8-[Bpy][NTf_2_] [174] and ZIF-8-[DBU-PEG][NTf_2_]_2_ [168], were obtained by dispersing MOFs in ILs. Recently, Gomes et al. [27] also selected ZIF-8 and Mg-MOF-74 as porous hosts and dispersed them in [P_66614_][NTf_2_], as shown in Fig. 17. The results showed that PLs were obtained by ZIF-8 but not by Mg-OF-74 because of its small pore apertures (3.4 Å) preventing the penetration of large long-chain cations. As a result, remarkable gas uptake performance (up to 150% more nitrogen and 100% more methane than pure IL) was realized at 303 K and 5 bar.

Overall, although examples of PLs for NH_3_ ab-adsorption have not been reported, the development of PLs for NH_3_ capture is attractive and promising from the perspective of fundamental research and practical applications. Importantly, the exciting results of CO_2_ capture realized by PLs reveal their promising application in efficient NH_3_ capture. At the same time, the aforementioned advanced ILs and various CPMs provide rich experience for designing novel PLs for NH_3_ capture.

## Emerging Membranes for NH_3_ Separation

Membrane separation is another potential option for NH_3_ capture because of its easy operation, low device occupancy, and energy saving [175–177]. Membrane separation can directly yield gaseous ammonia components without regeneration and has become increasingly attractive. However, unlike the extensive research on membranes for classical gases such as CO_2_, studies on NH_3_ capture using membranes are limited. Current research has primarily focused on the design of membrane materials to improve NH_3_ permeability and selectivity. One effective measure is to introduce interaction sites to enhance NH_3_ adsorption on the surfaces of membranes. Accelerating the NH_3_ diffusivity in the membrane by constructing transport pathways is another effective strategy. Table 5 displays the NH_3_ separation performances of representative membrane materials.

**Table 5:** NH_3_ separation performance of membrane materials

Membrane Material	P_NH3_(Barrer)	α_NH3/N2_	α_NH3/H2_	Condition^a^	References
PDMS	6551.9	27.5		21 °C	[179]
TPX	188.4	25.5			
LDPE	15	25			
ETFE	17.3	34.6			
PTFE	0.5	1.3			
FEP	2.5	2.5			
Hyflon AD40	17.2	3.0			
Hyflon AD60	41	3.0			
Teflon AF1600	228.8	2.9			
Teflon AF2400	1635.4	3.0			
SBI-26 (CH-cast)	473	591		20 °C,100 kPa	[182]
SBI-26 (THF-cast)	277	146			
POCE-PSS	612	50.6	50.1	25 °C,100 kPa	[181]
Pebax 1657	595.8	406.7	70.1	RT^b^,100 kPa	[184]
Nexar	496	566	88.6	RT^b^,100 kPa	[186, 187]
POI-GI-POSS-0	489	157.7	104	25 °C, 110 kPa	[189]
POI-GI-POSS-0.1wt%	716	477.3	88.4		
POI-GI-POSS-0.5wt%	841	467.2	25.8		
POI-GI-POSS-1.0wt%	1032	543.2	21.3		
POI-GI-POSS-2.0wt%	434	4.6	10.3		
POI-GI-POSS-5.0wt%	528	5.2	9.9		
POI-GI-POSS-8.0wt%	210	1.7	1.8		
ZIF-21^c^	25,910	35	12	RT^b^, ~ 141 kPa	[195]
MXene	18.4	24.6	14.53	100 kPa	[193]
PB/Au/AAO^d^	5.48	> 100	40	100 kPa	[194]
CA/PEG/MWCNTs-0	204	5		RT^b^,300 kPa	[201]
CA/PEG/MWCNTs-5wt%	2127	70.9			
CA/PEG/MWCNTs-10wt%	2390	95.6			
CA/PEG/MWCNTs-15wt%	17,957	4.3			
CA/PEG/MWCNTs-20wt%	21,017	2.2			
CA/PEG/MWCNTs-30wt%	24,612	1.1			

^a^Testig condition includes testing temperature and transmembrane pressure. ^b^RT represents the room temperature. ^c^The thickness of separation layer is ~ 15 μm. ^d^The thickness of separation layer is 15–50 nm

Polymeric membranes are the most typically reported NH_3_ separation membranes because of their high processability, in which gas permeation basically obeys the solution-diffusion mechanism. The NH_3_ transport properties of commercial cellulose acetate (CA) membranes were reported in 2006, and the results showed that the sorption process, dominated by hydrogen bond interactions between the NH_3_ molecules and membranes, played an important role in NH_3_ permeation [178]. Afterward, various polymers were exploited as membranes for NH_3_ separation, such as fluorinated polymers [179, 180]. However, these polymeric membranes showed great permeability but little selectivity and still suffered a trade-off effect between permeability and selectivity (called the Robeson upper bound).

To overcome the above challenges, researchers have proposed the design of polymeric membranes with NH_3_-interacted sites, such as sulfonated copolymers, to enhance the selective adsorption and solubility coefficients of ammonia. Phillip et al. [181] regulated the domain size of sulfonated block copolymers and the degree of crosslinking to affect NH_3_ separation performance. The results confirmed that the membrane designed using this strategy could retain high selectivity (mixed NH_3_/N_2_ selectivity > 90) compared to a Nafion membrane. Ansaloni et al. [182] further adjusted the membrane morphology of midblock-sulfonated pentablock ionomers (SBI-26 and SBI-52) by changing the type of casting solvent used to construct micro-domains conducive to NH_3_ separation. Recently, fluorinated sulfonic acid polymer/ceramic composite membranes with high thermal stability were developed, in which the acidic sulfonic groups on the polymer chains acted as NH_3_ sites, exhibiting NH_3_ separation performance with NH_3_ permeance of > 2.31 × 10^−6^ mol m^−2^ s^−1^ Pa^−1^, NH_3_/H_2_ separation factor of 90, and NH_3_/N_2_ of 800 at 50 °C in a mixed system [183].

**Fig. 18:**
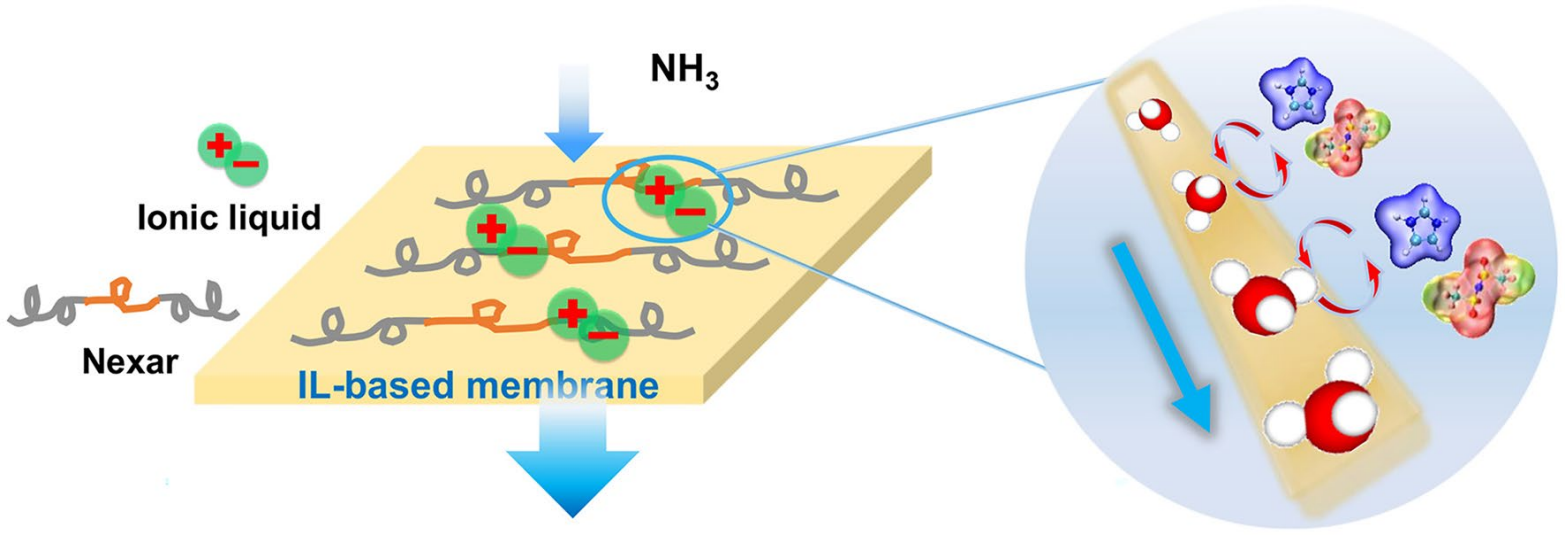
Schematic diagram of IL/Nexar hybrid membranes for NH_3_ separation (Reproduced with permission from Ref. [186]. Copyright 2021, Elsevier

In addition, incorporating NH_3_-interacted small molecules, such as ILs, into the polymer matrix is expected to improve the NH_3_ permeability and selectivity. As expected, the NH_3_ permeability was remarkably enhanced with increasing IL content, benefiting from the enhanced NH_3_ solubility [184, 185]. It is worth mentioning that the moderating interaction between membranes and NH_3_ plays an important role in increasing NH_3_ solubility, while excessively strong interactions also restrict NH_3_ diffusivity. Therefore, the selection of appropriate ILs, such as hydroxyl task-specific ILs, is crucial for preparing membranes with high permeances. The optimum NH_3_ permeability reached 3729.3 barrer with an NH_3_/N_2_ ideal selectivity of 1110.8, which are increases of 265.3% and 163.7%, respectively, compared to neat Pebax membrane. In addition, ILs enrich the ionic domains of block polymers to construct effective gas transport channels. The self-assembled NH_3_ transport channels induced by ILs and high NH_3_ affinity (Fig. 18) cooperatively promoted an increase in the NH_3_ diffusion and solubility coefficients, resulting in superior NH_3_ separation performance with an NH_3_ permeability of 3565 barrer and NH_3_/N_2_ and NH_3_/H_2_ selectivity as high as 1865 and 364, respectively [186, 187].

Effective strategies include constructing a transport channel and increasing the free volume of the membrane to promote gas transport/diffusivity and enhance ammonia separation. Wang et al. [188] first adopted a simulation method to verify the NH_3_ separation possibility of 2D-polyphthalocyanine (PPc) membranes with intrinsic pores. A high (H_2_/N_2_)/NH_3_ selectivity of 10^7^ was obtained at room temperature. Inspired by the above idea, Zaripov et al. [189] further prepared bulky agent octaglycidyl polyhedral oligomeric silsesquioxane (Gl-POSS) branched membranes, in which the disordered nanopores formed by the polymer segments greatly promoted NH_3_ transport. High ideal selectivity was achieved at Gl-POSS contents of 0.5–1 wt%. Subsequently, various membrane materials such as porous silica [190], ceramics [191], and zeolites [192], and other inorganic membranes, have been developed to achieve selective separation with the help of different pore sizes. However, unsatisfactory separation performance has encouraged researchers to further develop novel membranes to meet application requirements. Strategies combining porous properties and preferential adsorption have been proposed. For example, Petukhov et al. [193] prepared a 2D MXene membrane for NH_3_ separation in which acidic termination groups of interlayer galleries greatly promoted basic NH_3_ adsorption, and the increase of interlayer distance caused by vapor adsorption within MXene’s stacked structure also contributed to enhanced NH_3_ diffusivity. Similarly, ultra-thin Prussian blue (PB) analog membranes with high NH_3_ sorption capacity, pore channels (size of cavities < 0.3 nm), and high transformation ability of ammonia into NH_4_^+^ were prepared, achieving high NH_3_ permeance exceeding 0.3 m^3^ (STP) m^−2^ bar^−1^ h^−1^ and ultimate ideal NH_3_/H_2_ selectivity of 40 and NH_3_/N_2_ selectivity over 100 [194]. Furthermore, Wei et al. [195] coupled preferential adsorption and the seizing effect to prepare a ZIF-21 membrane with ~ 2.8 Å polar channel. Polar pores with limited apertures could efficiently seize NH_3_ molecules from gas mixtures. These two factors jointly promoted the ZIF-21 membrane to exhibit a high NH_3_ permeance of 1727 GPU with NH_3_/N_2_ and NH_3_/H_2_ ideal selectivities of 35 and 12, respectively. Considering the similarity between NH_3_ and H_2_O in terms of polarity and molecular size, Yu et al. [196, 197] developed an Na^+^-gated nanochannel membrane via a secondary growth method, which allowed small and polar NH_3_ molecules to permeate while blocking other non-polar and/or larger molecules, exhibiting remarkable selectivity (NH_3_/H_2_ > 4280 and NH_3_/N_2_ > 10,000 at 250 °C and 35 bar), excellent chemical stability, and long-term running stability.

Mixed matrix membranes (MMMs) have attracted increasing attention for NH_3_ separation in recent years because of the synergistic effect of both polymer and porous components [198–200]. Raza et al. [201] introduced carboxylic group-functionalized multiwall carbon nanotubes (COOH-MWCNTs) into a CA/PEG polymer matrix, which notably increased the permeability of NH_3_ and N_2_ owing to the enhanced voids and free volume. In addition, HKUST-1/PVDF MMMs were exploited by Cohen et al. [202] because HKUST-1 can bind with ammonia via Lewis acid–base interactions. Moreover, the HKUST-1 MMMs exhibited outstanding structural stability and maintained their ammonia capacity better than unstable powder under humid conditions. Our group [203] further combined HKUST-1 and protic IL [Bim][NTf_2_] to improve NH_3_ separation performance by employing hydrogen bond interactions. The optimal ternary MMM exhibited ideal NH_3_/N_2_ and NH_3_/H_2_ selectivities of 530.1 and 94.2, respectively. The optimal NH_3_ permeability reached up to 3680.0 barrer, which is 260% and 129% higher than those of the pristine Pebax membrane and Pebax/HKUST-1 MMM, respectively.

## Conclusions and Prospects

To effectively capture such hydrogen-rich, carbon-free, but highly corrosive molecules with triple the properties of energy, environment, and resources, great strides have been made in the development of advanced materials in the last decade. In this review, recent advances in NH_3_ capture materials, particularly those over the past 5 years, were briefly summarized. Major obstacles for specific applications, such as absorbents (functional solvents), adsorbents (porous solids), and membranes, were identified based on extensive studies. The interaction sites and transport pathways play a crucial role in improving NH_3_ capture performance. The potential application of the emerging hybrid technology, ab-adsorbents, using porous liquids as key capture materials, was also discussed. This review answers the question of how to connect advanced materials and NH_3_ capture technology via modulation of interaction sites and transport pathways.

However, for these NH_3_ capture technologies to be accepted as green strategies, the structure–property relationships between the materials and special parameters need to be further clarified, for which many challenges must be faced. In other words, there are worthwhile directions for researchers to further develop single/hybrid material designs and applications from either experimental or theoretical perspectives. The following aspects could be considered:

1. *Intelligent design & rational prediction* Designing novel materials and predicting their NH_3_ capture performance via a combination of theoretical calculations and experiments is highly desired. Various materials offer many possibilities for NH_3_ capture; however, relying solely on experimental methods normally requires a long time. An effective strategy for obtaining an optimal solution is to utilize computational artificial intelligence (AI)-assisted molecular design and high-throughput screening technologies. Specifically, the properties of existing ammonia capture materials can be analyzed and important structures for efficient NH_3_ capture can be extracted to build data- and mechanism-driven modes to further guide the development of high-performance materials. Furthermore, exploiting hybrid materials with complementary components, such as PLs, MOF@COF, and COF@MOFs, based on such recognition to further widen the variety of materials and obtain unforeseen structures has the potential to improve NH_3_ capture performance. Interfacial properties, such as compatibility, interaction synergy, and growth mechanism, of hybrid materials are also worth exploring for further development of novel materials. In addition, the determination of the synthesis conditions and process parameters with the assistance of mobile robotic chemists is the most promising method for shortening the research and development process*.*
2. *Excellent performance & high stability* The development of NH_3_ capture materials with high capacity, fast transport, and good stability remains challenging. Various functional materials have been developed based on the unique Lewis/Brønsted base and hydrogen bond formation properties of NH_3_ molecules, while most of them still suffer from low capacity, slow kinetics, and structural collapse. In addition, although many NH_3_ capture materials have been reported, breathing materials with a flexible nature (energy-saving synergistic adsorption–desorption) and dynamic properties (kinetic-induced non-equilibrium separation) are relatively limited. Therefore, the design of a robust system with both a high NH_3_ capacity and soft porosity is a worthwhile direction to explore for practical applications.
3. *Scale-up* The large-scale preparation of materials should be considered to meet the requirements of industrial applications. Although significant progress has been achieved, especially in advanced materials for NH_3_ capture, most current research is still limited to laboratories, and the yields of some materials are very small. Effective measures to boost scaled-up production and industrial applications need to be taken, such as screening inexpensive raw materials, simplifying synthesis steps, and using environmentally friendly synthetic methods. Specifically, expensive raw materials can be replaced with low-cost ones to synthesize various materials for NH_3_ capture using a one-step rather than multi-step method to synthesize materials to omit complex purification process, using green solvents, such as water, as much as possible such as water to avoid volatile organic solvents. In addition, optimizing flow diagrams for chemical processes, heat exchange networks, and performing energetic–environmental–economic assessments using process simulation software are expected to improve energy efficiency and reduce operating costs to accelerate the process of industrialization. In future, AI-assisted reaction simulations will be a powerful platform for exploring effective solutions to overcome the drawbacks of stepwise amplification and accelerate industrialization.
4. *Practical evaluation* The practical utilization conditions of various materials should be considered. Most current studies have investigated capture performance under ambient pressure in pure gas. However, the composition, pressure, and NH_3_ concentration of handling gases in real life are different, such as in the NH_3_ synthesis process (NH_3_/H_2_/N_2_, > 10 MPa, 10%–20% NH_3_), personal protective equipment (NH_3_/air, NH_3_ < 5000 ppm), and NH_3_ decomposition process (NH_3_/H_2_, NH_3_ < 0.1ppm). Therefore, the data obtained in existing studies are far from reflecting realistic conditions. Based on the above analysis, it is necessary to upgrade existing equipment and perform operando characterizations to further evaluate the actual performance and reveal mechanisms in the future. Specifically, existing equipment must be improved to match the handling conditions (pressure and composition) of product gases to obtain a more realistic evaluation. The design of the internals should be optimized to meet fluid mechanics requirements, thereby achieving excellent mass and heat transfer in the equipment. Operando characterizations should be performed under practical working conditions to track the ab/adsorption and desorption of NH_3_ molecules from various materials and to study possible structural changes under actual conditions to further reveal separation mechanisms [110, 204, 205].
5. *Integration process* Absorption–adsorption–membrane separation to develop an integrated technology to achieve self-adapting NH_3_ capture is a promising direction for future research. Because massive amounts of NH_3_-containing gases from various sources face different separation requirements, multi-process integration is more efficient and applicable than a single technology. A rational process design for integrated technology is expected to achieve material cost and energy consumption savings.

Overall, functional solvents, porous solids, porous liquids, and membranes are potential alternatives for NH_3_ capture. Although the road ahead is unknown, we firmly believe that various materials will become more competitive in the future through long-term and constant efforts.

## Abbreviations

AA: Acetamide
ACs: Activated carbons
[Bmim][SCN]: 1-Butyl-3-methylimidazolium thiocyanate
[Bmim][NTf_2_]: 1-Butyl-3-methylimidazolium bis(trifluoromethylsulfonyl)imide
[Bmim][Zn_2_Cl_5_]: 1-Butyl-3-methylimidazolium chlorozincate
[Bmim]_2_[Co(NCS)_4_]: 1-Butyl-3methylimidazolium tetraisothiocyanatocobaltate(II))
[Bmim][MeSO_3_]: 1-Butyl-3-methylimidazolium methanesulfonate
[Bmim]_2_[CuCl_4_]: Bis(1-butyl-3methyl imidazolium) copper tetrachloride
[Bmim]_2_[NiCl_4_]: Bis(1-butyl-3methyl imidazolium) nickel tetrachloride
[Bmim]_2_[SnCl_4_]: Bis(1-butyl-3methyl imidazolium) stannum tetrachloride
[Bpy][NTf_2_]: N-butyl pyridinium bis(trifluoromethyl sulfonyl)imide
[BOHmim][Zn_2_Cl_5_]: 1-(4-Hydroxy-butyl)-3-methylimidazolium chlorozincate
[Bim][NTf_2_]: 1-Butylimidazolium bis(trifluoromethylsulfonyl)imide
BBTA: 1H,5H-benzo(1,2d), (4,5-d′)bistriazole
BTC: 1,3,5-Benzenetricarboxylic acid
BTDD: Bis(1H-1,2,3triazolo[4,5-b],[4′,5′-i])dibenzo[1,4]dioxin
CPMs: Crystalline porous materials
COFs: Covalent organic frameworks
CIPMs: Conventional inorganic porous materials
CHBs: Cooperative hydrogen bonds
ChCl: Choline chloride
CA: Cellulose acetate
COOH-MWCNTs: Carboxylic group functionalized multiple-wall carbon nanotubes
[choline][NTf_2_]: Choline bis(trifluoromethylsulfonyl)imide
[CAM][Cl]: Carbamide chloride
DESs: Deep eutectic solvents
2D/3D materials: Two/three-dimensional materils
dodpdc: 4,4'-Dihydroxybiphenyl-3,3'-dicarboxylic acid
[DBU-PEG][NTf_2_]_2_: 8,8′ (3,6Dioxaoctane1,8diyl)bis(1,8Diazabicyclo[5.4.0]undec7en8ium) bis(trifluoromethylsulfonyl)imide)
[DMEA][Ac]: N,N-dimethylethanolammonium acetate
[EtOHim][NTf_2_]: 1-Hydroxyethyl-3-methyl bis(trifluoromethylsulfonyl)imide
[EtOHim][BF_4_]: 1-Hydroxyethyl-3-methyl tetrafluoroborate
[Emim][NTf_2_]: 1-Ethyl-3-methylimidazolium bis(trifluoromethyl sulfonyl)imide
[EtOHmim][NTf_2_]: 1-(2-Hydroxyethyl)-3-methylimidazolium bis(trifluoromethylsulfonyl)imide
[Emim][BF_4_]: 1-Ethyl-3-methylimidazolium tetrafluoroborate
[Emim][Ac]: 1-Ethyl-3-methylimidazolium acetate
[Emim][EtOSO_3_]: 1-Ethyl-3-methylimidazolium ethylsulfate
[Emim][SCN]: 1-Ethyl-3-methylimidazolium thiocyanate
[EtOHim][SCN]: 1-Hydroxyethyl-3-methyl thiocyanate
[EtOHmim][BF_4_]: 1-(2-Hydroxyethyl)-3-methylimidazolium tetrafluoroborate
[Eim][Li(NTf_2_)_2_]: 1-Ethylimidazolium lithium bi(bis(trifluoromethylsulfonyl)imide)
[Emim]_2_ [Co(NCS)_4_]: 1-Ethyl-3-methylimidazolium tetraisothiocyanatocobaltate(II))
EaCl: Ethylamine hydrochloride
EG: Ethylene glycol
[EtA][SCN]: Ethanolamine thiocyanate
EGDMA: Ethylene glycol dimethacrylate
ENIL: Encapsulated ionic liquid
FPMD: First-principles molecular dynamics
GCMC: Grand canonical Monte Carlo
Gly: Glycerol
Gl-POSS: Octaglycidyl polyhedral oligomeric silsesquioxane
HBAs: Hydrogen-bond acceptors
HBDs: Hydrogen-bond donors
HOFs: Hydrogen bond frameworks
HS: Hollow silica
HTCS: High throughput computational screening
[Hmim][BF_4_]: 1-Hexyl-3-methylimidazolium tetrafluoroborate
[Hmim]_2_ [Co(NCS)_4_]: 1-Hexyl-3-methylimidazolium tetraisothiocyanatocobaltate(II))
ILs: Ionic liquids
[Im][NO_3_]: Imidazolium nitrate
[Im][NTf_2_]: Imidazolium bis(trifluoromethylsulfonyl)imide
[Li-TEG][NTf_2_]: Lithium triethylene glycol bis(trifluoromethylsulfonyl)imide
MOPs: Metal–organic polyhedral
MOFs: Metal organic frameworks
MAA: N-methylacetamide
MMMs: Mixed matrix membranes
[2-Mim][NTf_2_]: 2-Methylimidazolium bis(trifluoromethylsulfonyl)imide
[2-Mim][Li(NTf_2_)_2_]: 2-Methylimidazolium lithium bi(bis(trifluoromethylsulfonyl)imide)
[MTEOA][MeOSO_3_]: Tris(2-hydroxyethyl)methylammonium methylsulfate
[MeOHim][NTf_2_]: 1-Hydroxymethyl-3-methyl bis(trifluoromethylsulfonyl)imide
[Me_2_C_2_^OH^N]Cl/: Dimethyl-di(2-hydroxyethyl)ammonium chloride
[MeC_3_^OH^N]Cl: Methyl-tri(2-hydroxyethyl)ammonium chloride
NA: Nicotinate
NIR: Near infrared spectroscopy
NMR: Nuclear magnetic resonance
OS: Organosilane
[Omim][BF_4_]: 1-Octyl-3-MethylImidazolium tetrafluoroborat
PLs: Porous liquids
PM2.5: 2.5-Micrometer particulate matter
POPs: Porous organic polymers
PhO: Phenol
PDAB-AA: Poly(divinylbenzene) acrylic acid
PIM-1-COOH: Polymers of Intrinsic Microporosity modified with carboxylic acid
PAA: Poly(amic acid)
PDF: Pair distribution function
PB: Prussian blue
PPc: Polyphthalocyanine
PDMS: Poly(dimethylsiloxane)
PVDF: Polyvinylidene difluoride
PEG: Polyethylene glycol
PIP: Porous ionic polymers
[P_66614_][NTf_2_]: Trihexyl(tetradecyl)phosphonium bis(trifluoromethylsulfonyl)imide
[Ph3ImH][NTf_2_]_2_: 1,3,5-Tri(imidazolium-1-yl) bi(bis(trifluoromethylsulfonyl)imide)
Res: Resorcinol
SOMP: Sulfonated and ordered mesoporous polymer
SCN: Thiocyanate
SILP: Supported ILs phase
SAXS: Small-angle X-ray scattering
TEG: Triethylene glycol
TA: Cobaltous thiocyanate
[1, 2, 3-TrizH_2_][NO_3_]_2_: 1, 2, 3-Triazolium nitrate
[1, 2, 3-TrizH_2_][CF_3_SO_3_]_2_: 1, 2, 3-Triazolium trifluoromethane sulfonate
